# Identification of Bicarbonate as a Trigger and Genes Involved with Extracellular DNA Export in Mycobacterial Biofilms

**DOI:** 10.1128/mBio.01597-16

**Published:** 2016-12-06

**Authors:** Sasha J. Rose, Luiz E. Bermudez

**Affiliations:** a Department of Biomedical Sciences, College of Veterinary Medicine, Oregon State University, Corvallis, Oregon, USA; b Department of Microbiology, College of Science, Oregon State University, Corvallis, Oregon, USA

## Abstract

Extracellular DNA (eDNA) is an integral biofilm matrix component of numerous pathogens, including nontuberculous mycobacteria (NTM). Cell lysis is the source of eDNA in certain bacteria, but the source of eDNA remains unidentified for NTM, as well as for other eDNA-containing bacterial species. In this study, conditions affecting eDNA export were examined, and genes involved with the eDNA export mechanism were identified. After a method for monitoring eDNA in real time in undisturbed biofilms was established, different conditions affecting eDNA were investigated. Bicarbonate positively influenced eDNA export in a pH-independent manner in *Mycobacterium avium*, *M. abscessus*, and *M. chelonae*. The surface-exposed proteome of *M. avium* in eDNA-containing biofilms revealed abundant carbonic anhydrases. Chemical inhibition of carbonic anhydrases with ethoxzolamide significantly reduced eDNA export. An unbiased transposon mutant library screen for eDNA export in *M. avium* identified many severely eDNA-attenuated mutants, including one not expressing a unique FtsK/SpoIIIE-like DNA-transporting pore, two with inactivation of carbonic anhydrases, and nine with inactivation of genes belonging to a unique genomic region, as well as numerous mutants involved in metabolism and energy production. Complementation of nine mutants that included the FtsK/SpoIIIE and carbonic anhydrase significantly restored eDNA export. Interestingly, several attenuated eDNA mutants have mutations in genes encoding proteins that were found with the surface proteomics, and many more mutations are localized in operons potentially encoding surface proteins. Collectively, our data strengthen the evidence of eDNA export being an active mechanism that is activated by the bacterium responding to bicarbonate.

## IMPORTANCE

Many bacteria contain extracellular DNA (eDNA) in their biofilm matrix, as it has various biological and physical functions. We recently reported that nontuberculous mycobacteria (NTM) can contain significant quantities of eDNA in their biofilms. In some bacteria, eDNA is derived from dead cells, but that does not appear to be the case for all eDNA-containing organisms, including NTM. In this study, we found that eDNA export in NTM is conditionally dependent on the molecules to which the bacteria are exposed and that bicarbonate positively influences eDNA export. We also identified genes and proteins important for eDNA export, which begins to piece together a description of a mechanism for eDNA. Better understanding of eDNA export can give new targets for the development of antivirulence drugs, which are attractive because resistance to classical antibiotics is currently a significant problem.

## INTRODUCTION

Nontuberculous mycobacteria (NTM) are opportunistic pathogens that are ubiquitous in the environment and are enriched in urban potable water systems (1). Even though NTM infections are not reported in many countries, analysis of smaller populations has shown that the prevalence of NTM infections has increased in recent years, with infections caused by *Mycobacterium avium* subsp. *hominissuis* and *M. intracellulare* being the most common (2–5). Though *M. avium* subsp. *hominissuis* infects individuals with immunosuppression through the gastrointestinal route and such infections often lead to disseminated disease, infections in immunocompetent patients are typically localized to the respiratory tract (1, 6). Underlying respiratory conditions such as cystic fibrosis, chronic obstructive pulmonary disease, and bronchiectasis do increase susceptibility for infection, but individuals who are otherwise healthy can also develop disease (5, 6).

How these infections establish and persist in the respiratory tract of immunocompetent patients is not completely understood, but growing evidence is supporting the hypothesis that, in addition to the well-described intracellular lifestyle in macrophages, NTM also colonize the airway through microaggregate and biofilm formation. Microscopy conducted on explanted lung sections from cystic fibrosis patients found *M. abscessus* biofilms on tissues, demonstrating the direct role of biofilms during these infections (7). In *M. avium* subsp. *hominissuis*, biofilm formation leads to better infection of epithelial cells *in vitro* and significantly influences respiratory infection *in vivo* (8). Furthermore, *M. avium* subsp. *hominissuis* forms mircoaggregates once in contact with respiratory epithelial cells and, once formed, the bacteria are more proficient at binding to and invading other cells (8–10). Surveilling macrophages that encounter *M. avium* subsp. *hominissuis* biofilm *in vitro* become hyperstimulated and undergo early, rapid apoptosis in a tumor necrosis alpha (TNF-α)-dependent manner (11), which could explain why the aggregates and biofilms are not cleared by the immune system.

The biofilm matrix of most bacteria is composed of exopolysaccharides (EPS), proteins, lipids, and nucleic acids (12). Mycobacteria form unique biofilms compared to most other biofilm-forming pathogens, in part because they do not produce EPS (13). Studies have identified some of the components of the unique mycobacterial biofilm matrix, including free mycolic acids (14, 15), glycopeptidolipids (16–19), and other lipid-containing molecules (20–22). Additionally, we recently reported the novel finding of extracellular DNA (eDNA) in both fast- and slow-growing NTM biofilms, including *M. avium* subsp. *hominissuis*, *M. abscessus*, *M. intracellulare*, and *M. chelonae*, among others (23). Treatment with DNase I reduced biofilm colonization *in vitro*, aided in removing established biofilms, and, in cotreatment with various clinically used antibiotics, reduced the *M. avium* subsp. *hominissuis* tolerance of the antimicrobials. Since that report, another study also recently described effective cotreatment of *M. chelonae* and *M. fortuitum* biofilms with DNase I and antimicrobial agents (24), further demonstrating the importance of eDNA in NTM biofilms.

eDNA has been found in the biofilm matrix of many bacterial genera, including numerous pathogens (25). In *Staphylococcus* spp., *Enterococcus* spp., and *Pseudomonas aeruginosa*, lytic events, including autolysis, H_2_O_2_-induced lysis, and prophage-mediated lysis, leading to eDNA release have been reported (26–30). In contrast to those findings, however, there are increasing numbers of reports describing nonlytic eDNA export in bacterial biofilms. Work with *Enterococcus faecalis* biofilms has shown convincing evidence for active eDNA secretion, including a lack of lysed cells, distinct eDNA morphologies between the fibrous eDNA and lysis-based DNA, bacteria possessing elevated membrane potentials, and no biochemical detection of intracellular components (31). Work with *Bacillus cereus* described early competence genes associated with eDNA export (32). A report on an aquatic bacterium, F8, detected distinct differences between the fibrous eDNA in the biofilm matrix and genomic DNA (33). In addition, reports of studies performed with *Streptococcus mutans* and *Acinetobacter baumannii* have identified eDNA in secreted membrane vesicles (34, 35). These reports of lysis-independent eDNA in biofilms agree with our finding of eDNA in *M. avium* subsp. *hominissuis*, which we did not observe in examining mass cell lysis microscopically or in monitoring CFU (23). Importantly, despite the growing evidence of the lysis-independent presence of eDNA in bacterial biofilms, the actual mechanism(s) of eDNA export remains unknown.

In this study, we investigated the triggers of eDNA export and began to elucidate a mechanism of lysis-independent eDNA export in *M. avium* subsp. *hominissuis*. We determined that exogenous bicarbonate induces eDNA export in a pH-independent manner, and by studying the biofilm surface proteome and screening transposon mutants, we have unveiled genes and gene products associated with eDNA export, including a DNA extrusion pore, carbonic anhydrases, and a novel genomic region.

## RESULTS

### Export of eDNA in *M. avium* is conditional.

**FIG 1:**
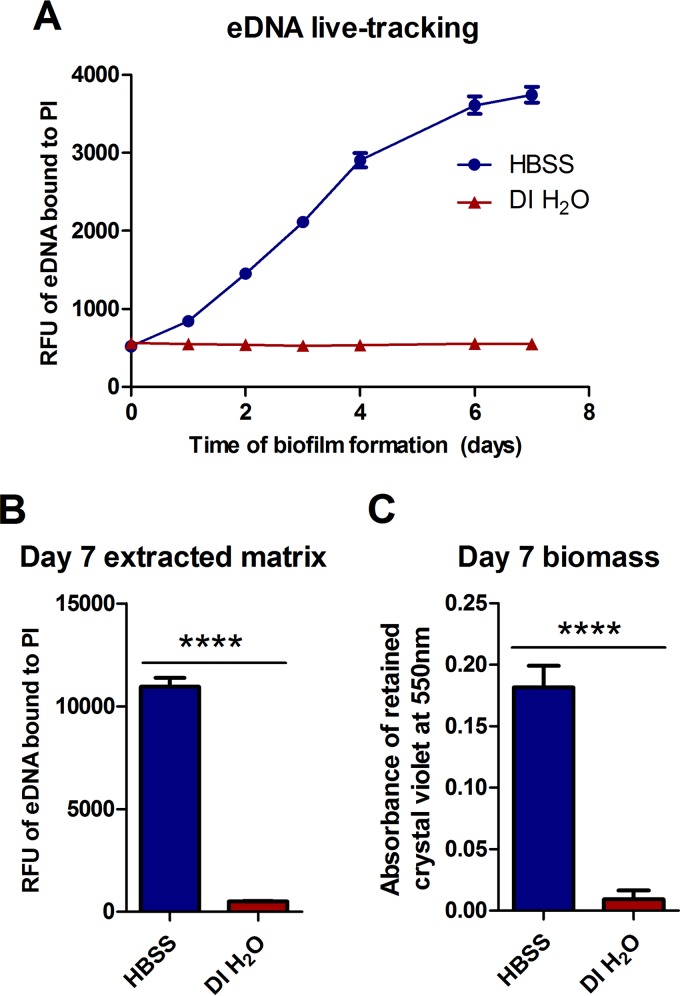
Conditional export of eDNA in *M. avium* subsp. *hominissuis* A5 biofilms. *M. avium* subsp. *hominissuis* biofilms were formed in HBSS and DI H_2_O, and eDNA was quantified. (A) Biofilms were formed with 3 µM cell-impermeative propidium iodide and incubated for a week. Undisrupted biofilms were fluorescently quantified over the time course to measure levels of eDNA bound to propidium iodide. Data points represent the average results from 6 separate biofilms ± standard deviations (SD). (B) Biofilms were formed without propidium iodide for 7 days, and then the biofilm matrix was extracted, filter sterilized, mixed with propidium iodide, and fluorescently read to quantify eDNA in the extract, as previously published (23). Bars represent the average results from 4 technical replicates ± SD. Data shown for both panel A and panel B are representative of results from three independent biological replicates. (C) Biomass was measured by analyzing the absorbance of retained and solubilized crystal violet at 550 nm that was bound to the attached biofilm. Bars represent the average results from 6 separate biofilms ± SD. Statistical comparisons: ****, *P* < 0.0001.

We previously reported that nontuberculous mycobacteria (NTM) export extracellular DNA when biofilms are formed in Hanks’ balanced salt solution (HBSS) (23). Importantly, no signs of massive cell lysis in *M. avium* subsp. *hominissuis* A5 biofilms were observed, which led to the hypothesis that a nonlytic mechanism could be responsible for eDNA in NTM biofilms. Due to this, we wanted to investigate variables that could affect eDNA export. We created a method for quantifying eDNA in undisturbed NTM biofilms as they form by coincubating a biofilm inoculum with the cell-impermeative fluorescent dye propidium iodide (PI). Agreeing with previous work (23), *M. avium* subsp. *hominissuis* A5 biofilms formed in HBSS exported a significant amount of eDNA over 7 days of biofilm formation (Fig. 1A). Interestingly, biofilms formed in deionized H_2_O (DI H_2_O) appeared to export very little or no eDNA throughout the time course (Fig. 1A). To ensure that this result was not an artifact of the assay, cell-free biofilm matrix was extracted from 7-day-old biofilms and then quantified fluorescently, as reported recently (23). Similarly to the live-tracking results, *M. avium* subsp. *hominissuis* A5 biofilms formed in DI H_2_O exported significantly less eDNA than the identical inoculum formed in HBSS (Fig. 1B). To assess if this conditional eDNA export affected biofilm robustness, biomass was analyzed using standard methods (36). In agreement with the lack of eDNA export, *M. avium* subsp. *hominissuis* A5 formed very weak biofilm in DI H_2_O (Fig. 1C). These stark differences suggest that eDNA in *M. avium* subsp. *hominissuis* A5 is conditionally exported and that a specific factor could trigger eDNA export and biofilm formation.

### Bicarbonate positively influences eDNA export in nontuberculous mycobacteria independently of pH.

**FIG 2:**
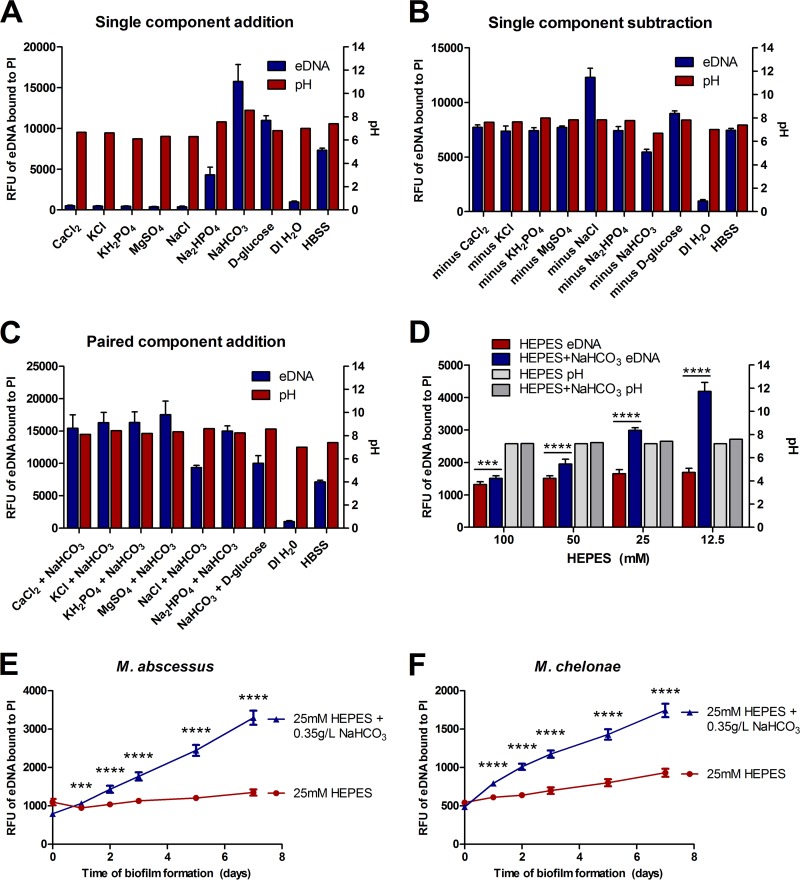
Bicarbonate influences eDNA export in NTM biofilms. Biofilm inoculums were made in different diluents with propidium iodide and incubated for 7 days, and eDNA levels were fluorescently measured over the time course. Data shown represent day 7 eDNA fluorescence and the pH of the respective solutions. Refer to Materials and Methods for concentrations of HBSS components. (A) Biofilms were formed in individual HBSS components in DI H_2_O. (B) Biofilms were formed in 7 of the 8 HBSS components, except for the listed component. (C) Biofilms were formed in individual HBSS components paired with bicarbonate in DI H_2_O. (D) Biofilms were formed in DI H_2_O buffered with different concentrations of HEPES with or without 0.35 g/liter bicarbonate. (E and F) Biofilms were formed in 25 mM HEPES with or without sodium bicarbonate in *M. abscessus* subsp. *abscessus* strain 19977 (E) and an environmental *M. chelonae* isolate (F) and subjected to live tracking for eDNA export analysis. For eDNA measurements, data shown are averages of results from 6 separate biofilms ± SD. Data shown are representative of results from two independent biological replicates. Statistical comparisons: ***, *P* < 0.001; ****, *P* < 0.0001.

Considering the significant differences in eDNA export between biofilms formed in HBSS and those formed in DI H_2_O, the components of our HBSS were further analyzed for their individual involvement with eDNA export in both an additive manner and a subtractive manner. Tracking eDNA in *M. avium* subsp. *hominissuis* A5, biofilm formed in single components in DI H_2_O, sodium phosphate partially restored eDNA to the levels exported in HBSS, and sodium bicarbonate and d-glucose both increased eDNA to a level higher than in HBSS (Fig. 2A, blue bars). Analyzing individual components in a subtractive manner, sodium bicarbonate is the only component that reduces eDNA levels to below the level exported in HBSS (Fig. 2B, blue bars). When either d-glucose or sodium chloride was removed, eDNA export was notably increased over HBSS levels, suggesting that those components impede the effect of bicarbonate. Comparing paired interactions between bicarbonate and the other individual components, d-glucose and sodium chloride reduced eDNA export compared to the other components (Fig. 2C, blue bars). All of the other possible pairs of components were tested, and the results agree with their respective single-component data (data not shown). Taken together, these data suggest that sodium bicarbonate positively influences eDNA export and is itself negatively affected by d-glucose and sodium chloride.

Since the pH of sodium bicarbonate by itself showed that it was the only ingredient more alkaline than HBSS (Fig. 2A, red bars), pH was analyzed initially (prior to inoculation and biofilm formation) for all other experimental conditions to assess the role of pH in eDNA export. There does not appear to have been a correlation between eDNA and pH, as there were many instances where the pH levels were similar for two samples but the levels of quantified eDNA differed significantly (Fig. 2B and C, red bars). To directly assess if sodium bicarbonate facilitates eDNA export due to pH, biofilms were formed with HEPES to keep the pH buffered (Fig. 2D). There was significantly more eDNA exported at all concentrations of HEPES with bicarbonate than was seen with solutions lacking bicarbonate (Fig. 2D). To determine if the bicarbonate anion was specifically what the bacteria were responding to under those conditions, different cations bound to bicarbonate were tested for their ability to trigger eDNA export in *M. avium* subsp. *hominissuis* A5. When buffered with HEPES, potassium bicarbonate and ammonium bicarbonate triggered eDNA export to levels comparable to those seen with sodium bicarbonate (see Fig. S1A to C in the supplemental material), independently of pH or CFU (Fig. S1D and E). There does appear to be a correlation between higher levels of eDNA export and lessening HEPES concentrations, but there were perhaps unseen chemical interactions between the HEPES and the various bicarbonates that could affect the activity of the bicarbonate anion with the bacteria.

To assess if bicarbonate influenced eDNA export in other NTM, *M. abscessus* and *M. chelonae*, which were previously shown to export substantial eDNA (23), were examined. In agreement with the results seen with *M. avium* subsp. *hominissuis* A5, bicarbonate buffered in HEPES elicited significantly more eDNA in *M. abscessus* and *M. chelonae* than HEPES alone (Fig. 2E and F, respectively). Taken together, the results demonstrate that bicarbonate triggers eDNA export in NTM and that this occurs independently of pH.

### Surface proteomics of bicarbonate-exposed biofilm reveal abundant carbonic anhydrases.

It has been previously described that the surface-exposed proteins of *M. avium* subsp. *hominissuis* vary depending on the environment that the bacteria are in, including different states of infection (37). To investigate the *M. avium* subsp. *hominissuis* surface-exposed and biofilm matrix proteins of *M. avium* subsp. *hominissuis* A5, mature HBSS-incubated biofilms were labeled using a cell-impermeative sulfo-NHS-LC-biotin reagent followed by streptavidin purification and mass spectrometric protein identification. Between two independent replicates, a total of 99 proteins were identified (see Table S1 in the supplemental material). Interestingly, a carbonic anhydrase (encoded by MAVA5_02375) was among the most abundant proteins identified (Table 1).

**TABLE 1:** The 10 most abundant surface-exposed proteins identified from a 7-day *M. avium* subsp. *hominissuis* A5 HBSS biofilm

Protein	A5 gene	Total spectrum count^a^
Wag31	MAVA5_10120	29
ATP synthase subunit beta	MAVA5_07195	27
Elongation factor Tu	MAVA5_19500	22
Superoxide dismutase	MAVA5_00855	22
Carbonic anhydrase	MAVA5_02375	20
Antigen 85-B	MAVA5_22455	18
Glutamine synthetase	MAVA5_09755	15
Catalase peroxidase	MAVA5_11495	14
MoxR protein	MAVA5_14195	11
DNA binding protein HU	MAVA5_16770	10

^a^ Total spectrum count data were determined by the use of a 95% peptide threshold and a 95% protein threshold with a two-peptide minimum level.

### Chemical inhibition of carbonic anhydrase reduces eDNA export.

**FIG 3:**
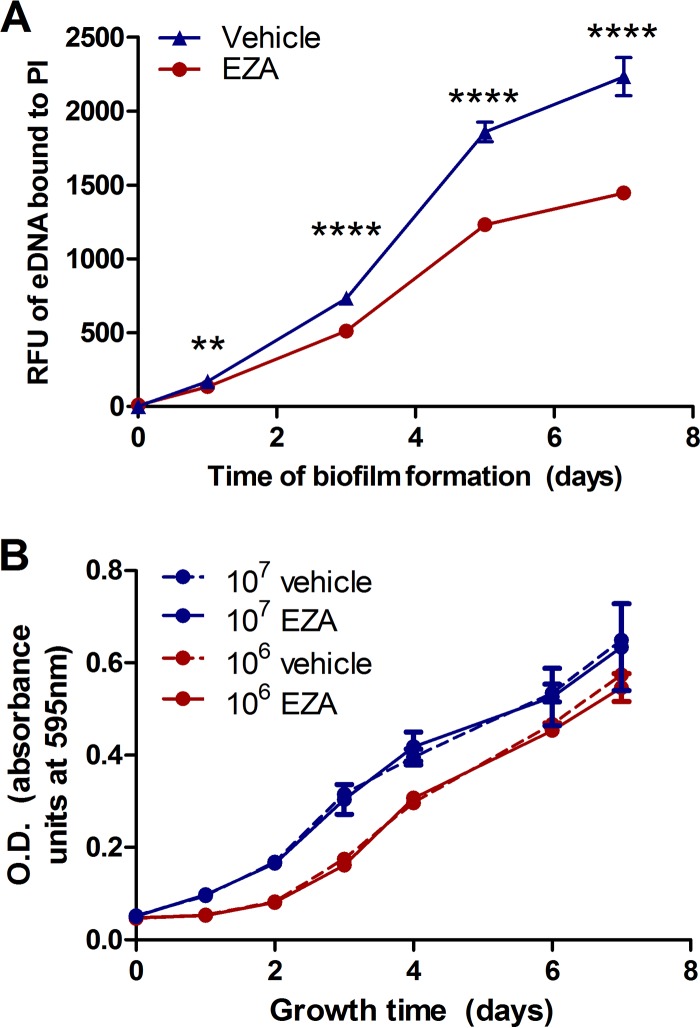
Carbonic anhydrase inhibition reduces eDNA levels in *M. avium* subsp. *hominissuis* A5 biofilms. (A) Biofilms were formed in HBSS either with 100 nM ethoxzolamide (EZA) from a 2 mM stock in 95% ethanol or with the equivalent dilution of ethanol vehicle, and eDNA levels were measured over a 7-day time course with propidium iodide. Data points represent the mean of results from 6 biofilms ± SD. (B) Inoculum levels of 10^7^ and 10^6^ *M. avium* subsp. *hominissuis* A5 cells freshly grown in either 100 nM EZA or vehicle were grown planktonically at 37°C for 7 days, and the optical density (OD) at 595 nm was measured daily. Statistical comparisons: **, *P* < 0.01; ****, *P* < 0.0001.

Due to the direct effect of bicarbonate on eDNA export and abundant surface-exposed carbonic anhydrases, chemical inhibition of carbonic anhydrase was explored to see it affects eDNA export. Ethoxzolamide (EZA) is a sulfonamide carbonic anhydrase inhibitor that has previously been demonstrated to have activity against *M. tuberculosis* carbonic anhydrases (38, 39). Biofilms were formed and subjected to live-tracking analysis with wild-type *M. avium* subsp. *hominissuis* strain A5 incubated in HBSS with either 100 nM EZA (from a 2 mM stock solution in 95% ethanol) or ethanol vehicle diluted similarly. EZA treatment significantly reduced eDNA export compared to treatment with the ethanol vehicle over the time course (Fig. 3A). To control for any indirect effects from the EZA that could affect bacterial viability or fitness, planktonic cultures were grown in the presence of 100 nM EZA or vehicle for 7 days. Planktonic bacteria formed at the same inoculum level as and a log lower than biofilms had no differences in growth rate (Fig. 3B), further supporting the results presented in Fig. 3A showing that EZA was working specifically against carbonic anhydrase(s) and was not indirectly affecting *M. avium* subsp. *hominissuis* A5.

### Transposon library screening for eDNA attenuation identified mutants putatively involved with bicarbonate, an eDNA secretion mechanism, and metabolism and a novel genomic region.

To elucidate genes involved with eDNA export and, potentially, the bicarbonate sensing mechanism, a mycobacteriophage-based transposon library was created in *M. avium* subsp. *hominissuis* A5 and 4,048 mutants were individually screened for eDNA export using the propidium iodide live-tracking method. From the screen, 173 mutants were cumulatively characterized as eDNA deficient and chosen for sequencing of transposon insertion locations. Among the 173 mutants, 158 gave definitive transposon locations when sequenced. A total of 126 of the 158 mutants had insertions directly interrupting genes, and 32 had insertions that were intergenic (Table S2). There were four instances where multiple mutants were identified with interruptions in the same gene (though the interruptions within the respective genes were all in unique sites): MAVA5_03165, encoding a GntR transcriptional regulator (mutants 19d12 and 26e9); MAVA5_05585, encoding a transcription-repair coupling factor (mutants 16e4 and 41c1); MAVA5_10275, encoding a metal-dependent hydrolase (mutants 26e12, 29b11, and 7d3); and MAVA5_10310, encoding a monooxygenase (mutants 43f8 and 5d3) (Table S2). In addition, there were 30 instances of mutants with mutations within a 10-gene proximity of those of other mutants (Table S2). Functional categories (downloaded from the J. Craig Venter Institute) were applied to the 126 mutants with directly interrupted genes, revealing metabolism and energy production (35 mutants, 28% of the total) and hypothetical (29 mutants, 23% of the total) to be the most abundant categories (Fig. S2 and Table S2). The large number of mutants involved with metabolism and energy production further suggests active processes involved with eDNA in *M. avium* subsp. *hominissuis* A5.

To quantitatively compare the levels of eDNA deficiency among the 126 sequenced mutants with directly interrupted genes, the day 7 eDNA fluorescent intensity (number of relative fluorescence units [RFU] of PI bound to eDNA) for each mutant was normalized to its starting optical density (Table S2). From this normalization, the mutant that was found to be most attenuated with respect to eDNA was 11e7 (encoded by MAVA5_03380), a FtsK/SpoIIIE-like DNA transporter (Table 2). 40c10 (MAVA5_19945), a carbonic anhydrase, was also among the most deficient (Table 2). There were mutants in two separate *S*-adenosylmethionine (SAM)-dependent methyltransferases as well as six hypothetical proteins among the most deficient mutants (Table 2). We assessed whether eDNA deficiency directly affects biofilm robustness by measuring the biomass of the HBSS-formed biofilms from the 15 most eDNA-deficient mutants against the wild-type bacterium. Agreeing with the results seen with the *M. avium* subsp. *hominissuis* A5 biofilm formed in DI H_2_O (Fig. 1C), the 15 most eDNA-deficient mutants from Table 2 formed significantly less biofilm than the wild type (Fig. S3).

**TABLE 2:** The 15 most eDNA-deficient mutants from *M. avium* subsp. *hominissuis* A5 Mmt7 library screen

Name	A5 gene	Encoded protein	eDNA/OD^a^
11e7	MAVA5_03380	FtsK/SpoIIIE	6,177
2e3	MAVA5_19115	Hypothetical (WXG100 domain)	6,432
29e7	MAVA5_10575	ABC transporter permease	6,619
42c3	MAVA5_13685	Hypothetical (Major facilitator superfamily domain)	6,654
39e5	MAVA5_20295	SAM-dependent methyltransferase	6,798
40c10	MAVA5_19945	Carbonic anhydrase	6,941
24d3	MAVA5_13900	Hypothetical protein (dehydrogenase domain)	7,105
41c1	MAVA5_05585	Transcription-repair coupling factor	7,184
36d7	MAVA5_05310	(2Fe−2S)-binding protein	7,469
40h2	MAVA5_10220	Hypothetical protein (MaoC dehydrogenase domain)	7,581
1e5	MAVA5_10615	SAM-dependent methyltransferase	7,871
36f9	MAVA5_13185	Oxidoreductase	7,932
35c4	MAVA5_01245	Hypothetical protein (19 kDa mycobacterial antigen domain)	7,960
39d10	MAVA5_13215	Cytochrome P450	8,000
14g4	MAVA5_07205	Hypothetical protein (Domain of unknown function)	8,077

^a^ The eDNA/optical density (OD) value for each sample was calculated by normalizing the fluorescence reading for day 7 RFU bound to PI to the starting OD of the respective sample.

The genome of *M. avium* subsp. *hominissuis* A5 was recently sequenced by our laboratory (GenBank accession no. GCA_000696715.1). The *M. avium* subsp. *hominissuis* A5 genome is roughly 500 kbp smaller than the *M. avium* subsp. *hominissuis* 104 reference genome and contains only small amounts of genetic material that *M. avium* subsp. *hominissuis* 104 is missing. Notably, there is an ~50-kbp genomic region (MAVA5_10140 through MAVA5_10380, Table S3) flanked by a tRNA. Nine eDNA-deficient mutants had genes located within this genomic region: 23d4 (MAVA5_10205, encoding an N5,N10-methylene tetrahydromethanopterin reductase); 40h2 (MAVA5_10220, encoding an a hypothetical protein); 7d3, 26E12, and 29b11, three mutants with mutations in different locations in MAVA5_10275, encoding a metal-dependent hydrolase; 37h6 (MAVA5_10295, encoding a flavin-binding monooxygenase); 43f8 and 5d3, two mutants with mutations in different locations in MAVA5_10310, encoding a 4-hydroxyacetophenone monooxygenase; and 37d4 (MAV_10315, encoding a cytochrome P450 protein).

**FIG 4:**
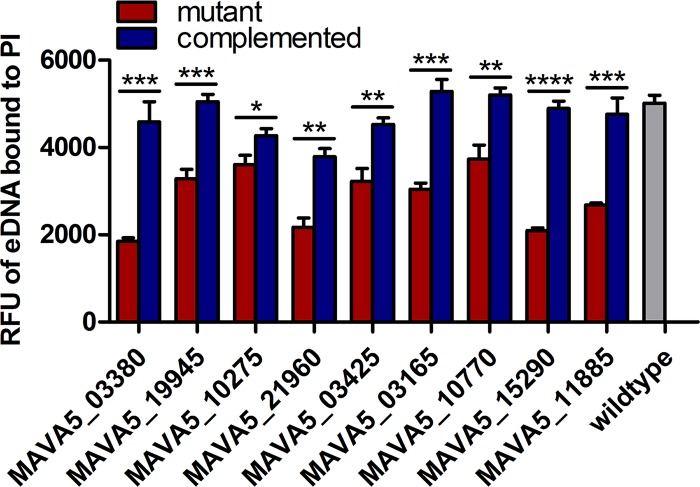
Complementation of eDNA-deficient mutants. Nine eDNA-deficient mutants were selected for complementation of the respective genes by the use of the integrative pMV306-Apr plasmid. Bacteria were grown in 7H9 broth with OADC containing no antibiotic (wild type), kanamycin (deficient mutants), or kanamycin and apramycin (complemented mutants). Biofilms were formed with propidium iodide, and eDNA was measured over a time course. Bars represent averages ± SD at day 7 of results from three separate biofilms that were grown separately in broth beforehand as well. Data shown are representative of results from at least two independent biological replicates. Statistical comparisons: *, *P* < 0.05; **, *P* < 0.01; ***, *P* < 0.001; ****, *P* < 0.0001.

To further confirm that eDNA-deficient mutants were directly related to eDNA export, nine mutants were chosen to complement a functional copy of the respective mutated gene along with up to 500 bp upstream of the gene into the integrative pMV306-Apr plasmid. A diverse set of mutants were selected for complementation and included the FtsK/SpoIIIE pore (encoded by MAVA5_03380), a carbonic anhydrase (MAVA5_19945), a metal-dependent hydrolase from the 50-kbp region (MAVA5_10275), a membrane protein (MAVA5_21960), TetR and GntR transcriptional regulators (MAVA5_03425 and MAVA5_03165, respectively), a precorrin-3B synthase (MAVA5_10770), and two hypotheticals (MAVA5_15290 and MAVA5_11885). Every complemented mutant significantly restored eDNA export compared to its noncomplemented counterpart (Fig. 4), further demonstrating the direct role of genes found in the eDNA transposon screen in eDNA regulation and export.

### Connection between eDNA-exporting surface proteome and eDNA-deficient mutants.

To corroborate the identifications of the proteins in the bicarbonate-exposed biofilm surface proteome with eDNA export, the identified proteins were analyzed against the sequenced eDNA-deficient mutants. In total, 44 of the 99 genes corresponding to identified surface proteins were in close proximity (5 genes) to identified eDNA mutants (Table 3). An additional 12 genes corresponding to identified proteins were located within 10 genes of eDNA-deficient mutants (data not shown). Five identified proteins match actual eDNA-deficient mutants, two are directly overhanging with eDNA-deficient mutants, and 24 are encoded by genes located in potential operons associated with eDNA-deficient mutants (Table 3). Interestingly, there were seven groupings of adjacent genes corresponding to identified proteins that are located in close proximity to eDNA-deficient mutants (Table 3). Collectively, the results suggest that a significant number of proteins encoded by genes important for eDNA in *M. avium* subsp. *hominissuis* A5 are localized extracellularly in these biofilms.

**TABLE 3:** Overlap of identified biofilm surface proteins with sequenced eDNA-deficient mutants

Gene of identified biofilm protein	eDNA-deficient mutant(s) in close proximity to gene of identified biofilm protein (MAVA5_XXXXX)	Exact proximity of identified protein from deficient mutant^a^	Adjacent identified proteins?
MAVA5_00730	23a3 (_00710)	4 genes downstream	
MAVA5_00855	11g6 (_00880)	5 genes away	
MAVA5_00885	11g6 (_00880)	1 gene away	
MAVA5_01985	1h11 (_01975)	2 genes away	
MAVA5_02995	13d3 (_03020)	5 genes downstream	
MAVA5_03155	19d12/26e9 (both _03165)	2 genes away	
MAVA5_03760	9b11 (_03755)	24 bp downstream	
MAVA5_03945	25d12 (_03920–_03925)	4 genes downstream	Yes
MAVA5_03950	25d12 (_03920–_03925)	5 genes downstream	Yes
MAVA5_04770	30e5 (_04775)	Overhanging	
MAVA5_05225	36h9 (_05210)	3 genes downstream	
MAVA5_05665	45e4 (_05645)	4 genes upstream	
MAVA5_05780	12h10 (_05775)	1 gene away	
MAVA5_07180	14g4 (_07205)	5 genes upstream	Yes
MAVA5_07185	14g4 (_07205)	4 genes upstream	Yes
MAVA5_07190	14g4 (_07205)	3 genes upstream	Yes
MAVA5_07195	14g4 (_07205)	2 genes upstream	Yes
MAVA5_09565	18b11 (_09590)	5 genes downstream	Yes
MAVA5_09570	18b11 (_09590)	4 genes downstream	Yes
MAVA5_09575	18b11 (_09590)	3 genes downstream	Yes
MAVA5_09755	41e1 (_09740)	3 genes away	
MAVA5_11495	46a1 (_11495)	Same gene	
MAVA5_11885	16g11 (_11885)	Same gene	
MAVA5_12085	6g5 (_12060)	5 genes away	
MAVA5_12110	30e6/44b10 (_12130/_12135)	4/5 genes away	
MAVA5_12185	34g9 (_12185–_12190)	Directly upstream	
MAVA5_13360	7b10 (_13345)	3 genes away	
MAVA5_13390	7b5 (_13410)	4 genes away	
MAVA5_14395	6f4 (_14420)	5 genes away	Yes
MAVA5_14400	6f4 (_14420)	4 genes away	Yes
MAVA5_16150	7e2 (_16155)	23 bp upstream	
MAVA5_16825	13f3 (_16800)	5 genes away	
MAVA5_19500	5d9 (_19495)	1 gene upstream	Yes
MAVA5_19510	5d9 (_19495)	3 genes upstream	Yes
MAVA5_19940	40c10 (_19945)	1 gene upstream	
MAVA5_20590	11d3 (_20565)	5 genes away	
MAVA5_21610	29c4 (_21610)	Same gene	Yes
MAVA5_21615	29c4 (_21610)	Overhanging	Yes
MAVA5_21640	29f12 (_21635)	1 gene downstream	Yes
MAVA5_21645	29f12 (_21635)	2 genes downstream	Yes
MAVA5_21745	26a3 (_21745)	Same gene	
MAVA5_21745	4c2 (_21735)	2 genes away	
MAVA5_22265	31g1 (_22270)	1 gene downstream	
MAVA5_22890	45c6 (_22890)	Same gene	

^a^ Downstream and upstream, all genes within the given proximity region were in the same orientation; away, genes within proximity region were in different orientations.

## DISCUSSION

The ability of nontuberculous mycobacteria (NTM) to form biofilm creates challenges for treatment efficacy and host clearance (7, 8, 10, 11). We recently reported that NTM contain extracellular DNA (eDNA) in their biofilm matrix that significantly contributes to colonization, persistence, and drug tolerance of these biofilms, suggesting a novel antivirulence target for NTM (23). Here, we described bicarbonate as a trigger for eDNA export in *M. avium* and identified genes and extracellular proteins that are involved with the mechanism(s) of eDNA export. Work performed with other eDNA-producing organisms found elevated membrane potentials (31), early competence genes (32), and secreted membrane vesicles containing eDNA (34, 35), but the genes and proteins involved with the actual mechanisms remain largely unknown. The method presented in this work for monitoring eDNA as it is exported allowed us to screen a large transposon library to identify genes associated with eDNA. Multiple mutations found in ATPases, ABC transporters, transcriptional regulators, and energy production and metabolism genes strengthen the idea of active processes involved with eDNA export. To validate the direct role of the genes found in the transposon screen with eDNA, we complemented nine eDNA-deficient mutants comprising a variety of different eDNA-deficient mutants. Testing the mutants against their complemented strains for eDNA export, all nine significantly restored eDNA levels. Note that not all nine were completely restored to wild-type levels. For the few complemented mutants that did not match wild-type levels, there could be multiple reasons for that outcome. The full function of these proteins could be dependent on the presence of nearby proteins and/or regulators, which the distant location of the integrative pMV306 plasmid could interfere with. Another possibility is that there was just not the same number of bacteria in the biofilm as was seen with the wild type. All biofilm inoculums for these experiments were equalized via optical density, but CFU levels were not confirmed.

Host organisms contain elevated carbon dioxide and bicarbonate concentrations compared to environmental sources due to respiration and the bicarbonate buffering system, and pathogens have developed mechanisms to sense these molecules. Epithelial cells of the respiratory tract secrete bicarbonate in airway surface fluid, which respiratory pathogens can access and interact with. *Cryptococcus*, *Bacillus*, and *Streptococcus* species respond to carbon dioxide to regulate gene expression (40–42). The AraC transcriptional regulators RegA in *Citrobacter rodentium*, PerA in *Enterococcus faecalis*, and ToxT in *Vibrio cholerae* directly respond to bicarbonate stimulation (43–45). Interestingly, *V. cholerae* was able to overcome the need for bicarbonate under carbon dioxide-enriched conditions for a ToxT response, but only while shaken (43). We have formed *M. avium* subsp. *hominissuis* A5 biofilms without bicarbonate under carbon dioxide-enriched conditions, but only in our static biofilm model, which did not yield notable eDNA export (data not shown). It would be interesting to adapt our biofilm model to a condition where we can more thoroughly infuse carbon dioxide into the biofilms, to see if this can stimulate eDNA export in the absence of bicarbonate. Unfortunately, we did not find any AraC mutants in our transposon screen; however, nine mutations were found in a unique genomic region in *M. avium* subsp. *hominissuis* A5 that does contain an AraC regulator gene (MAVA5_10195), and eDNA-deficient mutant genes were identified two and four genes downstream from this regulator gene. *M. tuberculosis* contains six AraC regulators, but, interestingly, Rv1395 is the only homolog to MAVA5_10195 (which on its own is unique among the annotated *M. avium* AraC regulator genes). A Rv1395 mutant was attenuated during respiratory infection of mice using a signature-tagged mutagenesis screen (46). Ongoing work in our laboratory is being performed to look at the genes that this AraC is influencing in both *M. avium* subsp. *hominissuis* A5 and *M. tuberculosis*.

One of the most abundant proteins identified with the extracellular biofilm proteomics was a DNA binding protein, HU (encoded by MAVA5_16770). Similar DNA binding proteins have been found extracellularly within other eDNA-containing biofilms (47, 48). Antisera targeting DNA binding proteins effectively disrupted eDNA architecture and biofilm integrity in various pathogens (47, 48). In our experience, treatment with trypsin destabilized *M. avium* subsp. *hominissuis* A5 biofilms and released eDNA into the biofilm supernatant (unpublished data), arguing that a protein component is participating with eDNA in these biofilms. Extracellular DNA binding proteins have also been shown to bind to host cells (49–51) and stimulate inflammatory responses from macrophages (52). *M. avium* subsp. *hominissuis* A5 elicited a robust proinflammatory response from macrophages that were exposed to biofilm (11), and the extracellular DNA binding protein HU could have contributed to that role. Further work will be needed to determine the specific function of MAVA5_16770.

Due to the abundance of the surface-exposed carbonic anhydrase encoded by MAVA5_02375 in the *M. avium* subsp. *hominissuis* A5 biofilm, we investigated eDNA export while using the carbonic anhydrase inhibitor ethoxzolamide. This sulfonamide has been described previously to have activity against *M. tuberculosis* carbonic anhydrases (38, 39). Even though we observed a significant reduction of the level of eDNA under conditions of coincubation with EZA, complete inhibition of eDNA was not achieved, which could have been due to multiple reasons. There are at least four carbonic anhydrase genes annotated in the *M. avium* subsp. *hominissuis* A5 genome, and perhaps EZA did not inhibit all of them, inhibition was temporary, or EZA could not access all of the functioning carbonic anhydrases. This agrees with the results seen with carbonic anhydrase eDNA-deficient mutants 40c10 (encoded by MAVA5_19945) and 20e5 (MAVA5_22765) found in our library screen: although significant eDNA deficiency was seen, complete elimination of eDNA was not observed. Recent work has shown that in *M. tuberculosis*, carbonic anhydrase inhibition with EZA interrupts both the PhoPR regulatory system and ESX-1 secretion (39). This is interesting, because the ESX-1 secretion system has been shown to be necessary for intracellular *M. tuberculosis* to permeabilize the phagosomal membrane and allow DNA exported in the phagosome to access cytosolic DNA receptors (53, 54). The *M. avium* subsp. *hominissuis* A5 genome does not contain a complete ESX-1 region but does contain ESX-2 through ESX-5 (unpublished data). Our screen did find eDNA-deficient mutants located in ESX-2 (23a3, MAVA5_00710), ESX-3 (8c10, MAVA5_21435), and ESX-5 (39f5, MAVA5_12405). The direct roles that these ESX systems have in eDNA export, if any, need to be further studied.

The most eDNA-deficient mutant found in our screen was 11e7, with a mutation in MAVA5_03380, encoding an FtsK/SpoIIIE-like DNA translocation protein. These proteins were first described in *Escherichia coli* to localize on the divisome during cell division and translocate copied chromosomal DNA into the daughter cell (55). Mycobacteria contain numerous genes that are annotated as encoding FtsK/SpoIIIE. Even though 11e7 was the most deficient mutant, eDNA was still observed. There could be multiple proteins responsible for eDNA translocation. Analyzing all of the genes encoding FtsK/SpoIIIE proteins in *M. avium* subsp. *hominissuis* A5, the only one that is homologous to MAVA5_03380 is MAVA5_10375, which is located in the unique 50-kbp region also identified as important for eDNA due to deficient mutants identified from it. We did not find a MAVA5_10375 mutant in our screen, but since this gene has not been directly studied, it is unknown if it is essential for growth, and our screen was limited to nonessential genes. The ligation-mediated PCR (LMPCR) method was very successful at sequencing mutants (158 of 173) but did not work for all mutants. We PCR screened the 15 eDNA-deficient mutants that did not work with LMPCR via regular PCR for intact MAVA5_10375, as well as for MAVA5_10195 (described earlier) and all the other annotated FtsK/SpoIIIE genes in the *M. avium* subsp. *hominissuis* A5 genome, and did not find any mutants. We are currently investigating the contribution, if any, of MAVA5_10375 to eDNA.

Identifying nine mutants deficient in eDNA that have mutations in genes located in the unique 50-kbp genomic region was remarkable. In our initial report on mycobacterial eDNA (23), the NTM species that exported notably more eDNA than others, close to the levels measured in *M. avium* subsp. *hominissuis* A5, were *M. abscessus* and *M. chelonae*. Furthermore, we demonstrated in the current work that these two mycobacterial species export eDNA when stimulated by bicarbonate, strengthening the idea that the mechanism of eDNA export in *M. avium* subsp. *hominissuis* A5 could be shared by other mycobacteria. When we first found this unique genomic region in *M. avium* subsp. *hominissuis* A5, we analyzed it against other mycobacteria and found it mainly in various strains in *M. abscessus*, *M. chelonae*, *M. avium*, and *M. intracellulare*. The GC content of this region (64%) matches that of *M. abscessus* and *M. chelonae* but is different from that of *M. avium* and *M. intracellulare* (both 69%). This suggests that the region could have been introduced horizontally into the *M. avium* complex from the *M. abscessus*/*M. chelonae* complex. Flanking the region is a tRNA, which furthers the possibility of horizontal transfer. Though we did not identify deficient mutants in AraC (encoded by MAVA5_10195) or FtsK/SpoIIIE (MAVA5_10375) in this region in our screen, their potential involvement with eDNA cannot be excluded. Further work on this interesting region will help determine the specific role it has in bicarbonate sensing and/or eDNA export.

## MATERIALS AND METHODS

### Bacterial strains and growth.

*Mycobacterium avium* subsp. *hominis. suis* strain A5 (*M. avium* subsp. *hominissuis* A5) was originally isolated from the blood of an AIDS patient. *M. abscessus* subsp. *abscessus* strain 19977 was obtained from the American Type Culture Collection. The *M. chelonae* isolate used is an environmental strain from Corvallis, OR, that was isolated by our laboratory and used previously (23). Unless otherwise noted, bacteria for experiments were grown and maintained on Middlebrook 7H10 agar supplemented with 10% oleic acid, albumin, dextrose, and catalase (OADC; Hardy Diagnostics, Santa Maria, CA) at 37°C.

### Biofilm formation.

Static biofilms were formed as described previously (11), with minor modifications. Briefly, a 10^9^ CFU/ml suspension was prepared in deionized H_2_O (DI H_2_O) from log-phase bacteria grown on 7H10 agar. After allowing 10 min for the clumps to sediment, the top half of the suspension was transferred and adjusted by diluting a sample of the suspension 1:10 and then assessing visual turbidity using a McFarland 1 standard (3 × 10^8^ CFU/ml). Once adjusted, biofilm inoculums were formed by mixing 10% (vol/vol) of the 3 × 10^9^ suspension with 90% (vol/vol) of an appropriate diluent. Biofilms were incubated at room temperature for up to 7 days for experimentation.

### Live tracking of eDNA export.

Upon biofilm formation, propidium iodide was added to the inoculum to reach a concentation of 3 µM from a 20 mM stock in dimethyl sulfoxide (DMSO). The fluorescence of propidium iodide bound to eDNA was quantified at wavelengths of 535 and 620 nm with an Infinite F200 microplate reader (Tecan, Männedorf, Switzerland). Readings were taken at time zero (upon plate inoculation) and then over a 7-day time course.

### HBSS component addition and subtraction.

The formulation of HBSS with calcium and magnesium (Corning, Corning, NY) used had the following components: anhydrous CaCl_2_, 0.14 g/liter; KCl, 0.4 g/liter; KH_2_PO_4_, 0.06 g/liter; anhydrous MgSO_4_, 0.0977 g/liter; NaCl, 8 g/liter; anhydrous Na_2_HPO_4_, 0.0477 g/liter; NaHCO_3_, 0.35 g/liter; d-glucose, 1 g/liter. Stock solutions (10×) of each of the 8 ingredients were made individually in DI H_2_O. For individual ingredient additive experiments, 10% (vol/vol) of the 3 × 10^9^ bacterial suspension (prepared as described above) was mixed with 10% (vol/vol) of a single 10× component, 80% (vol/vol) DI H_2_O, and propidium iodide to reach 3 µM. Paired-component additive experiments were set up similarly, with the exception that two 10× components were added and only 70% (vol/vol) DI H_2_O was used. For subtractive experiments, 10% (vol/vol) of the 3 × 10^9^ bacterial suspension was mixed with different combinations of 7 of the 8 components (all at 10% [vol/vol]) and 20% (vol/vol) DI H_2_O. Real-time quantification of eDNA export was fluorescently measured as described above.

### Biotinylation and purification of surface-exposed and biofilm matrix proteins.

The procedures used for biotin labeling and streptavidin purification from *M. avium* subsp. *hominissuis* A5 biofilms were adapted from the method described by McNamara et al. (37). Biofilms (7 days old) were resuspended with a cell scraper and then centrifuged at 2,000 × *g* for 15 min. Supernatant was removed, and pellets were gently resuspended in HBSS containing 1 mg of EZ-Link sulfo-NHS-LC biotin (Thermo Fisher, Waltham, MA). Samples were incubated at 4°C for 30 min using rotation. Glycine was added to reach a concentration of 10 mM for 10 min to quench the excess reagent. Samples were washed three times in HBSS via centrifugation and resuspended in a guanidinium lysis buffer (6 M guanidine HCl–10 mM EDTA–2 mM EGTA–0.1% Tween-20–phosphate-buffered saline [PBS], adjusted to pH 7.2) with 0.2 ml of 0.1-mm-diameter glass beads and homogenized. Samples were centrifuged at 10,000 × *g* for 10 min, and supernatant was collected and filtered through a 0.2-µm-pore-size syringe filter. C1 streptavidin-coupled Dynabeads (Thermo, Fisher) (80 µl) were added to each sample and incubated, using rotation, at room temperature for 1 h. The beads were then washed (using a magnetic separation stand) twice with the guanidinium lysis buffer and then twice with PBS–0.05% Tween-20. Beads were resuspended in 35 µl of 0.1% SDS–H_2_O and incubated at 65°C for 10 min. An equal volume of 2× Laemmli buffer was added, and samples were incubated at 95°C for 10 min and then 72°C for 10 min. Samples were centrifuged for 2 min at 200 × *g* to pellet the beads, and then the supernatant was collected for downstream analysis.

### Protein purification and mass spectrometry.

Biotin-labeled protein samples were submitted to electrophoresis until they traveled 1 cm into a 12% polyacrylamide gel, and then the whole 1-cm piece was excised. Samples were prepared and digested using protease Max surfactant (Promega, Madison, WI) in combination with trypsin Gold (Promega) following the manufacturer protocol, with minor modifications, including multiplying all volumes by 5 prior to the digestion step, and the digestion was carried out with 100 µl of protease Max containing 300 ng of trypsin for the 10-min rehydration followed by overlaying with 500 µl of protease Max. Digested samples were submitted to the Environmental Health Science Center Mass Spectrometry Facility at Oregon State University for mass spectrometric protein identification, following their established protocols. Briefly, samples were further purified with a C_18_ column and analyzed with an LTQ-FT mass spectrometer (Thermo) coupled with a NanoAcquity ultraperformance liquid chromatography (UPLC) system (Waters). Proteome Discoverer v 1.3.0, Mascot v 2.3, and Scaffold were used for data analysis. The UniProt_MAV104 database was used for database searches. Proteomics data are displayed as total spectrum count with the following parameters: 95% peptide threshold (using the Scaffold Local false-discovery-rate [FDR] algorithm) and 95% protein threshold (with a two-peptide minimum for protein identity).

### MycomarT7 transduction and transposon library creation.

MycomarT7 (Mmt7) is a temperature-sensitive transposon-containing phagemid (56) that was kindly provided by Eric Rubin (Harvard T.H. Chan School of Public Health, Boston, MA). Mmt7 stock was propagated and titers were determined using *M. smegmatis* strain mc^2^155 as previously described (57). To transduce *M. avium* subsp. *hominissuis* A5, bacteria were first grown at 37°C in 7H9 broth supplemented with 10% OADC and 0.1% Tween-80 with shaking at 200 rpm. Bacteria were washed 2 times with 37°C MP buffer (150 mM NaCl, 50 mM [pH 7.5] Tris-HCl, 10 mM Mg_2_SO_4_, 2 mM CaCl_2_) and then resuspended in 37°C MP buffer and diluted to 6 × 10^8^ CFU/ml using visual turbidity. A 5-ml volume of this suspension (3 × 10^9^) was infected with Mmt7 at a multiplicity of infection (MOI) of 2. Bacteria were transduced at 37°C for 4 h with intermittent mixing. After transduction, aliquots were plated on 7H10 agar containing 400 µg/ml kanamycin to obtain individual transposon mutants. Sixty kanamycin-resistant colonies were screened for the presence of Mmt7 with PCR, and all colonies tested were positive for the presence of Mmt7, even after replating and confirming with PCR a second time.

### Transposon library screen for eDNA export.

A total of 4,048 transposon mutants were placed into 250 μl of 7H9 supplemented with OADC and 400 μg/ml kanamycin and grown for 5 days in deep, round-bottom 96-well plates at 37°C using 210 rpm of rotation. Plates were centrifuged at 2,000 × *g* for 15 min to pellet bacteria. The supernatant was removed, and pellets were resuspended into HBSS containing 3 μM propidium iodide and transferred into flat-bottom polystyrene 96-well plates. The initial optical density of the plates was measured with a 595-nm filter. eDNA export was measured as described earlier.

### Transposon location identification.

Transposon insertion locations on mutants of interest were identified utilizing a previously reported ligation-mediated PCR (LMPCR) technique, with minor modifications (58, 59). Cell lysate was produced by resuspending bacteria into DI H_2_O, adding 0.2 ml of 0.1-mm-diameter glass beads, and homogenizing. Samples were centrifuged at 10,000 × *g* for 2 min to pellet cell debris, and then the DNA was purified from the supernatant using a DNA Clean and Concentrate kit (Zymo Research, Irvine, CA) following the manufacturer’s protocol. DNA (100 to 150 ng) was subjected to single digestion with SalI or double digestion with BamHI and BglII (all enzymes were Thermo fast-digest enzymes) for 20 min at 37°C. LMPCR adapters were made for both SalI (Salgd+Salpt adapter; see Table S4 in the supplemental material) and BamHI/BglII (Salgd+Bampt adapter; Table S4) by mixing equal molar amounts of each oligonucleotide with 1× Taq DNase buffer plus MgCl_2_ and ligating by decreasing the temperature from 80°C to 4°C over the course of 1 h. Digested DNA (25 ng) was ligated with 0.5 μl of freshly prepared 100 µM adapters using T4 DNase ligase. A 0.5-μl volume of the product of this ligation was used as the template in the LMPCR reaction, which was performed using a Fidelitaq system (Affymetrix, Santa Clara, CA). Two sets of primers were used for each sample that shared the reverse primer (complementary to the adapter) and had the forward primer at either the inverted repeat of the transposon or 150 bp upstream into the transposon (Table S4). The LMPCR reaction was performed using 97°C for 7 min, 40 cycles of 97°C for 30 s, 58°C for 1 min, and 72°C for 1 min 45 s, and then a final step of 72°C for 10 min. PCR products were visualized using ethidium bromide and agarose gel electrophoresis, following standard protocols. Bands of interest (preferably, the l50-bp-larger band for each sample that contains transposon sequence) were excised, purified, and submitted to the Center for Genome Research and Biocomputing at Oregon State University for DNA sequencing.

### Complementation of mutants.

Genes and their native promoters were complemented via the use of the integrative pMV306 plasmid as described before (60) with minor modifications. First, an apramycin resistance gene was cloned into the pMV306 plasmid, producing the pMV306-Apr plasmid. Genes of interest and up to 500 bp of upstream DNA were cloned into pMV306-Apr using primers listed in Table S4. Electrocompetent *M. avium* subsp. *hominissuis* A5 mutant cells were prepared by washing plate-grown bacteria 5 times via centrifugation at 2,000 × *g* and 4°C for 15 min in a chilled 10% glycerol–0.1% Tween-80 solution, followed by resuspension in a small volume of chilled 10% glycerol to concentrate the competent cells. PCR and constructs verified by double restriction digestion were electroporated into the respective *M. avium* subsp. *hominissuis* A5 electrocompetent mutants in a 0.2-cm-gap-width cuvette using 2,500 V, 1,000 Ω, and 25 µF. Bacteria were recovered in 7H9 broth supplemented with 10% OADC overnight, followed by plating on 7H10 plates containing 400 µg/ml kanamycin and 400 µg/ml apramycin. Colonies were PCR screened using the same primers as described above to confirm positive clones.

### Statistics.

Statistical comparisons between groups were made using a two-tailed homoscedastic *t* test. Detailed information about data presentation and *P* value definitions are listed in each applicable figure legend. Graphical outputs were created with GraphPad Prism software.

## SUPPLEMENTAL MATERIAL

Figure S1  Analysis of eDNA export with various bicarbonate-bound cations. (A to C) To assess whether the bicarbonate anions specifically in sodium bicarbonate, potassium bicarbonate, and ammonium bicarbonate were tested side by side, sodium bicarbonate at 0.35 g/liter (the concentration of sodium bicarbonate in HBSS) was used in all experiments. Biofilm inoculums were buffered in HEPES at various concentrations. (D) eDNA export was measured in real time over 7 days, but data shown represent day 7 eDNA export levels, for comparative purposes. Bars represent averages of results from 6 separate biofilms ± SD. Data shown are representative of results from two independent biological replicates. (E) The starting pH of these different conditions was recorded to confirm the effectiveness of the HEPES buffering. At day 7 of biofilm formation, duplicate wells were resuspended 50× via pipetting, serially diluted, and plated to assess CFU count differences between the different bicarbonate cations. Statistical comparisons (all compared to HEPES control): *, *P* < 0.05; **, *P* < 0.01; ***, *P* < 0.001. Download Figure S1, TIF file, 3.4 MB

Figure S2  Functional analysis of eDNA-deficient *M. avium* subsp. *hominissuis* A5 mutants. Biofilms were formed with 4,048 individual clones from a *M. avium* subsp. *hominissuis* A5 transposon library, and eDNA was quantified over the time course. Functional roles were assigned to the 126 sequenced eDNA-deficient mutants with directly interrupted genes and are shown as percentages of the total 126 mutants (see Table S2 for individual mutant functional assignments). Download Figure S2, TIF file, 0.9 MB

Figure S3  Biofilm formation ability of 15 most eDNA-deficient mutants. Biofilms were formed in HBSS from the 15 most eDNA-deficient mutants from Table 2 and compared with wild-type *M. avium* subsp. *hominissuis* A5 biofilms. Biomass was measured by analyzing the absorbance of retained and solubilized crystal violet that was bound to the attached biofilm. Bars represent the average results from 4 separate biofilms ± SD. Data shown are representative of results from two independent biological replicates. Statistical comparisons versus wild-type data: **, *P* < 0.01, ***, *P* < 0.001, ****, *P* < 0.0001. Download Figure S3, TIF file, 2.8 MB

Table S1  All identified surface-exposed proteins from 7-day-old *M. avium* subsp. *hominissuis* A5 biofilm. Table S1, DOCX file, 0.01 MB

Table S2  All sequenced eDNA-deficient A5 transposon mutants. Table S2, DOCX file, 0.02 MB

Table S3  Unique *M. avium* subsp. *hominissuis* strain A5 genomic region that nine eDNA-deficient mutants were located within. Table S3, DOCX file, 0.01 MB

Table S4  Primers used in this study. Table S4, DOCX file, 0.01 MB
