# Supplementary figures and images for: Identification of Bicarbonate as a Trigger and Genes Involved with Extracellular DNA Export in Mycobacterial Biofilms
Source: mBio. 2016 Dec 6;7(6):e01597-16. doi: 10.1128/mBio.01597-16 (PMC5142616; doi:10.1128/mBio.01597-16)

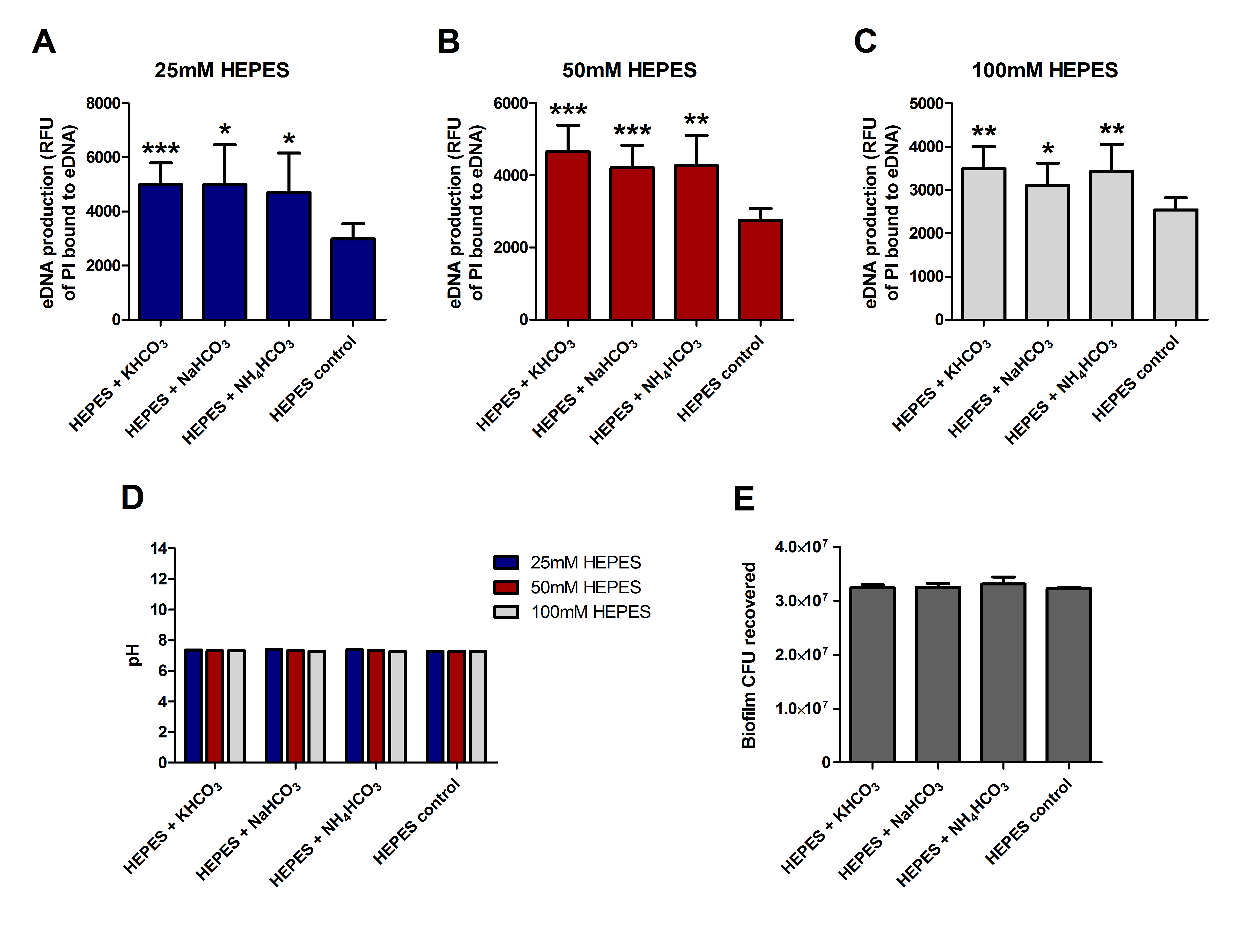

Supplement: Figure S1 — Analysis of eDNA export with various bicarbonate-bound cations. (A to C) To assess whether the bicarbonate anions specifically in sodium bicarbonate, potassium bicarbonate, and ammonium bicarbonate were tested side by side, sodium bicarbonate at 0.35 g/liter (the concentration of sodium bicarbonate in HBSS) was used in all experiments. Biofilm inoculums were buffered in HEPES at various concentrations. (D) eDNA export was measured in real time over 7 days, but data shown represent day 7 eDNA export levels, for comparative purposes. Bars represent averages of results from 6 separate biofilms ± SD. Data shown are representative of results from two independent biological replicates. (E) The starting pH of these different conditions was recorded to confirm the effectiveness of the HEPES buffering. At day 7 of biofilm formation, duplicate wells were resuspended 50× via pipetting, serially diluted, and plated to assess CFU count differences between the different bicarbonate cations. Statistical comparisons (all compared to HEPES control): *, P < 0.05; **, P < 0.01; ***, P < 0.001. Download [file mbo006163096sf1.tif]

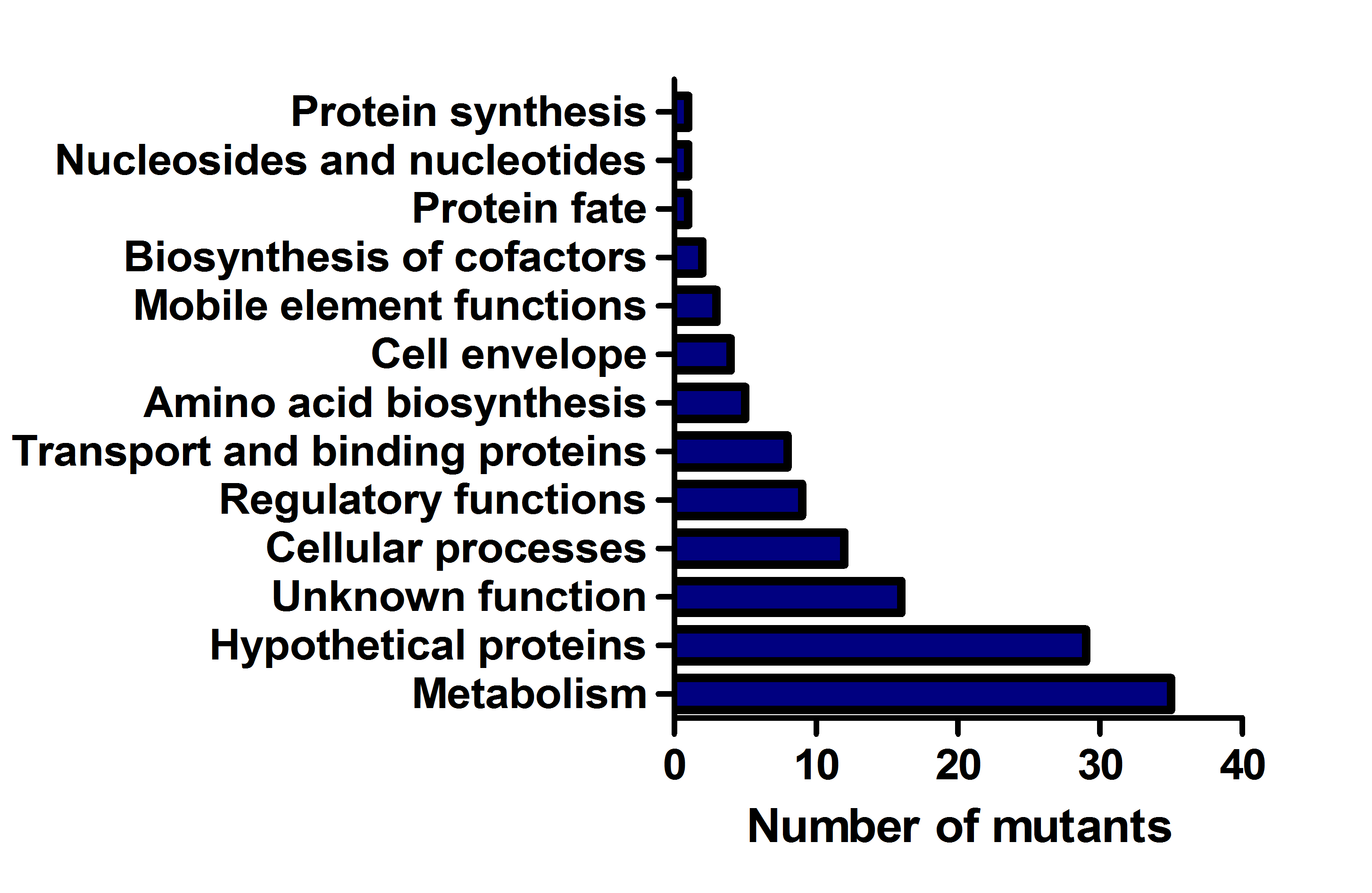

Supplement: Figure S2 — Functional analysis of eDNA-deficient M. avium subsp. hominissuis A5 mutants. Biofilms were formed with 4,048 individual clones from a M. avium subsp. hominissuis A5 transposon library, and eDNA was quantified over the time course. Functional roles were assigned to the 126 sequenced eDNA-deficient mutants with directly interrupted genes and are shown as percentages of the total 126 mutants (see Table S2 for individual mutant functional assignments). Download [file mbo006163096sf2.tif]

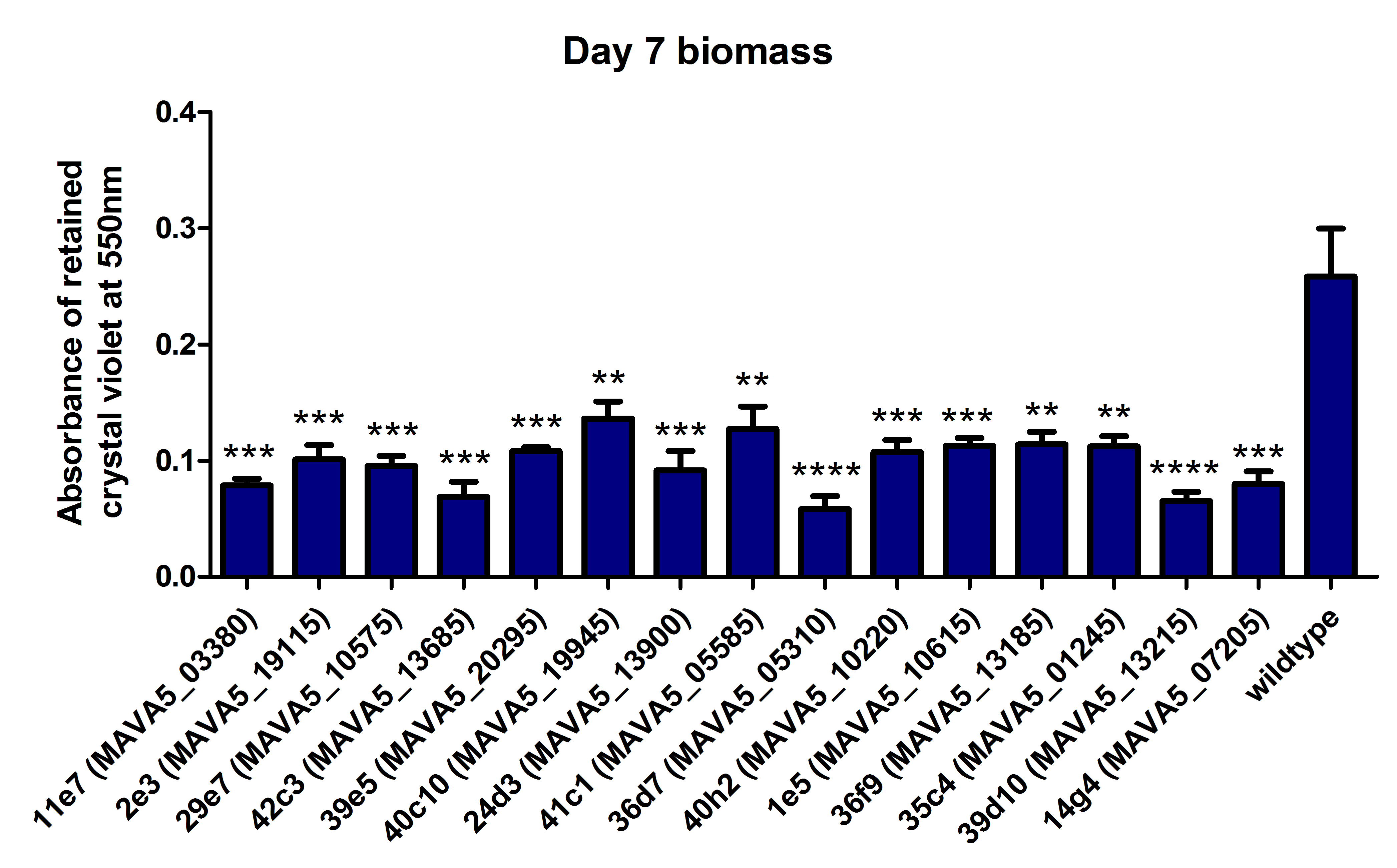

Supplement: Figure S3 — Biofilm formation ability of 15 most eDNA-deficient mutants. Biofilms were formed in HBSS from the 15 most eDNA-deficient mutants from Table 2 and compared with wild-type M. avium subsp. hominissuis A5 biofilms. Biomass was measured by analyzing the absorbance of retained and solubilized crystal violet that was bound to the attached biofilm. Bars represent the average results from 4 separate biofilms ± SD. Data shown are representative of results from two independent biological replicates. Statistical comparisons versus wild-type data: **, P < 0.01, ***, P < 0.001, ****, P < 0.0001. Download [file mbo006163096sf3.tif]
