# Supplementary material for: Identification of Bicarbonate as a Trigger and Genes Involved with Extracellular DNA Export in Mycobacterial Biofilms
Source: mBio. 2016 Dec 6;7(6):e01597-16. doi: 10.1128/mBio.01597-16 (PMC5142616; doi:10.1128/mBio.01597-16)
Supplement: Table S1 — All identified surface-exposed proteins from 7-day-old M. avium subsp. hominissuis A5 biofilm. [file mbo006163096st1.docx]

Supplemental Table 1. All identified surface-exposed proteins from 7 day old MAH A5 biofilm

| Description | MAH A5 gene | Total Spectrum Count^a^ |
| --- | --- | --- |
| Wag31 protein | MAVA5_10120 | 29 |
| ATP synthase subunit beta | MAVA5_07195 | 27 |
| elongation factor Tu | MAVA5_19500 | 22 |
| superoxide dismutase | MAVA5_00855 | 22 |
| carbonic anhydrase | MAVA5_02375 | 20 |
| Antigen 85-B | MAVA5_22455 | 18 |
| glutamine synthetase | MAVA5_09755 | 15 |
| Catalase-peroxidase | MAVA5_11495 | 14 |
| MoxR protein | MAVA5_14195 | 11 |
| DNA-binding protein HU | MAVA5_16770 | 10 |
| malate dehydrogenase | MAVA5_06685 | 10 |
| 35kd antigen | MAVA5_15895 | 9 |
| oxidoreductase | MAVA5_21645 | 9 |
| peroxisomal multifuctional enzyme type 2 | MAVA5_22265 | 9 |
| putative uncharacterized protein | MAVA5_00730 | 9 |
| universal stress protein family protein | MAVA5_13390 | 9 |
| ATP synthase subunit alpha | MAVA5_07185 | 9 |
| electron transfer flavoprotein | MAVA5_16965 | 8 |
| cluster of Retinol dehydrogenase 13 | MAVA5_05545 | 8 |
| DNA-directed RNA polymerase subunit alpha | MAVA5_03950 | 8 |
| putative uncharacterized protein | MAVA5_08060 | 8 |
| acetyl-CoA acetyltransferase | MAVA5_05780 | 8 |
| glycerol-3-phosphate dehydrogenase | MAVA5_19940 | 8 |
| fructose-bisphosphate aldolase class-I | MAVA5_22890 | 7 |
| heparin binding hemagglutinin hbha | MAVA5_20430 | 7 |
| malate synthase G | MAVA5_12185 | 6 |
| transaldolase | MAVA5_14335 | 6 |
| two component transcriptional regulator trcr | MAVA5_05665 | 5 |
| immunogenic protein MPT64 | MAVA5_18240 | 5 |
| 30S ribosomal protein S7 | MAVA5_19510 | 5 |
| isocitrate lyase | MAVA5_11710 | 5 |
| transcriptional regulator, Crp/Fnr family protein | MAVA5_01985 | 5 |
| thiol peroxidase | MAVA5_11660 | 5 |
| PPE family protein | MAVA5_12310 | 5 |
| ABC transporter, ATP-binding protein | MAVA5_19575 | 5 |
| 30kDa chaperonin 2 | MAVA5_20590 | 4 |
| acetyl-/propionyl-coenzyme A carboxylase | MAVA5_18840 | 4 |
| putative thiosulfate sulfurtransferase | MAVA5_18830 | 4 |
| 10 kDa chaperonin | MAVA5_19365 | 4 |
| prephenate dehydratase | MAVA5_00885 | 4 |
| acetyl-CoA acetyltransferase | MAVA5_21640 | 4 |
| Lsr2 protein | MAVA5_02325 | 4 |
| putative molybdenum cofactor synthesis protein | MAVA5_05225 | 4 |
| glutamine synthetase, type I | MAVA5_09735 | 4 |
| phosphotriesterase-like protein | MAVA5_21745 | 4 |
| ATPase family protein (AAA+) | MAVA5_01770 | 3 |
| dienelactone hydrolase family protein | MAVA5_10805 | 3 |
| enoyl-CoA hydratase | MAVA5_19725 | 3 |
| uncharacterized oxidoreductase | MAVA5_16675 | 3 |
| ModD protein | MAVA5_12085 | 3 |
| 30S ribosomal protein S5 | MAVA5_03700 | 3 |
| putative acyl-CoA dehydrogenase | MAVA5_09940 | 3 |
| enoyl-CoA hydratase | MAVA5_16150 | 3 |
| 50S ribosomal protein L3 | MAVA5_03585 | 3 |
| short chain dehydrogenase | MAVA5_12110 | 3 |
| ATPase family protein (AAA+) | MAVA5_01770 | 3 |
| D-3-phosphoglycerate dehydrogenase | MAVA5_16825 | 3 |
| aldo/keto reductase | MAVA5_18060 | 3 |
| major membrane protein 1 | MAVA5_08920 | 3 |
| glyceraldehyde-3-phosphate dehydrogenase | MAVA5_14400 | 3 |
| MihF protein | MAVA5_14605 | 3 |
| saccharopine dehydrogenase | MAVA5_08150 | 3 |
| HpcH/HpaI aldolase/citrate lyase family protein | MAVA5_17490 | 3 |
| acyl carrier protein | MAVA5_09575 | 3 |
| ATP synthase gamma chain | MAVA5_07190 | 3 |
| PPE family protein | MAVA5_18930 | 3 |
| putative uncharacterized protein | MAVA5_16665 | 3 |
| nitroreductase family protein | MAVA5_19205 | 3 |
| putative uncharacterized protein | MAVA5_20165 | 3 |
| [NADH]enoyl-[acyl-carrier-protein] reductase | MAVA5_14170 | 3 |
| MaoC family protein | MAVA5_19685 | 3 |
| gamma-glutamyl phosphate reductase | MAVA5_08270 | 2 |
| ATP synthase subunit b-delta | MAVA5_07180 | 2 |
| aspartate transaminase | MAVA5_01615 | 2 |
| phosphoribosylformyglycinamidine synthase I | MAVA5_03155 | 2 |
| 30S ribosomal protein S2 | MAVA5_16425 | 2 |
| aconitate hydratase 1 | MAVA5_14215 | 2 |
| HIT domain protein | MAVA5_02995 | 2 |
| naphthoate synthase | MAVA5_20035 | 2 |
| 3-oxoacyl-[acyl-carrier-protein] synthase 1 | MAVA5_09570 | 2 |
| branched-chain-amino-acid aminotransferase | MAVA5_09815 | 2 |
| quinone oxidoreductase | MAVA5_14320 | 2 |
| transketolase | MAVA5_14330 | 2 |
| succinate dehydrogenase | MAVA5_21615 | 2 |
| 30S ribosomal protein S4 | MAVA5_03945 | 2 |
| hydrolase, alpha/beta hydrolase fold family protein | MAVA5_06485 | 2 |
| 6-phosphogluconolactonase | MAVA5_14350 | 2 |
| methyltransferase type 12 | MAVA5_13360 | 2 |
| putatuve uncharacterized protein | MAVA5_04770 | 2 |
| triosephosphate isomerase | MAVA5_14390 | 2 |
| succinate dehydrogenase | MAVA5_21610 | 2 |
| phosphoribosylaminoimidazole carboxylase | MAVA5_18785 | 2 |
| cluster of 2-oxoacyl-[acyl-carrier-protein] | MAVA5_09565 | 2 |
| nitrogen regulatory protein P-II | MAVA5_16575 | 2 |
| putative uncharacterized protein | MAVA5_03760 | 2 |
| 60 kDa chaperonin 1 | MAVA5_19360 | 2 |
| putative uncharacterized protein | MAVA5_05150 | 2 |
| LprG protein | MAVA5_14525 | 2 |
| phosphoglycerate kinase | MAVA5_14395 | 2 |

^a^Total spectrum count determined by 95% peptide threshold and 95% protein threshold with a two peptide minimum.
