# Supplementary material for: Identification of Bicarbonate as a Trigger and Genes Involved with Extracellular DNA Export in Mycobacterial Biofilms
Source: mBio. 2016 Dec 6;7(6):e01597-16. doi: 10.1128/mBio.01597-16 (PMC5142616; doi:10.1128/mBio.01597-16)
Supplement: Table S2 — All sequenced eDNA-deficient A5 transposon mutants. [file mbo006163096st2.docx]

Supplemental Table 2. All sequenced eDNA deficient A5 transposon mutants.

| **Mutant** | **d7/O.D.** | **A5 gene** | **Description** | **JCVI functional category** |
| --- | --- | --- | --- | --- |
| 3f4 | 10,261 | MAVA5_00150 | conserved hypothetical protein | Hypothetical proteins |
| 18c7 | 11,217 | MAVA5_00260 | leucyl-tRNA synthetase | Protein synthesis |
| 33c5 | 13,970 | intergenic | 20bp upstream from MAVA5_00320 | N/A |
| 23a3 | 11,238 | MAVA5_00710 | Hypothetical protein | Hypothetical proteins |
| 11g6 | 9,615 | MAVA5_00880 | conserved hypothetical protein | Hypothetical proteins |
| 35c4 | 7,960 | MAVA5_01245 | lipoprotein LpqH | Unknown function |
| 34c12 | 10,730 | MAVA5_01250 | metallo-beta-lactamase superfamily protein | Unknown function |
| 17g10 | 9,598 | MAVA5_01565 | dihydrodipicolinate reductase, N-terminus domain protein | Amino acid biosynthesis |
| 29b4 | 12,617 | MAVA5_01820 | formate dehydrogenase, alpha subunit | Energy metabolism |
| 28f5 | 10,995 | MAVA5_02140 | conserved hypothetical protein | Hypothetical proteins |
| 27g3 | 12,966 | MAVA5_02215 | hypoxanthine phosphoribosyltransferase | Nucleosides, and nucleotides |
| 45f8 | 9,153 | MAVA5_02520 | putative flavin reductase | Fatty acid and phospholipid metabolism |
| 24e5 | 12,324 | MAVA5_02785 | thiamine pyrophosphate enzyme | Unknown function |
| 42f6 | 10,863 | intergenic | 40bp upstream from MAVA5_02875 | N/A |
| 8d10 | 9,211 | intergenic | 7bp upstream from MAVA5_02950 | N/A |
| 13d3 | 19,340 | MAVA5_03020 | cytochrome P450 51 | Fatty acid and phospholipid metabolism |
| 19d12 | 8,090 | MAVA5_03165 | transcriptional regulator, GntR family protein | Regulatory functions |
| 26e9 | 9,351 | MAVA5_03165 | transcriptional regulator, GntR family protein | Regulatory functions |
| 27c6 | 11,684 | MAVA5_03295 | phosphate ABC transporter, permease protein PstA | Transport and binding proteins |
| 11e7 | 6,177 | MAVA5_03380 | cell divisionftsk/spoiiie | Cellular processes |
| 2f5 | 8,209 | MAVA5_03410 | conserved hypothetical protein | Hypothetical proteins |
| 38h12 | 8,469 | MAVA5_03425 | putative transcriptional regulator | Regulatory functions |
| 3d1 | 15,062 | MAVA5_03655 | conserved hypothetical protein | Hypothetical proteins |
| 9b11 | 10,560 | MAVA5_03755 | carbohydrate kinase, FGGY family | Unclassified |
| 25d12 | 11,285 | intergenic | 103bp upstream from MAVA5_03920 | N/A |
| 13g9 | 14,096 | MAVA5_04105 | transposase | Mobile element functions |
| 29d11 | 9,106 | MAVA5_04290 | 4-carboxymuconolactone decarboxylase domain protein | Energy metabolism |
| 30e5 | 8,420 | MAVA5_04775 | conserved hypothetical protein | Hypothetical proteins |
| 4e12 | 12,293 | intergenic | non-CDS region. Nearest is MAVA5_04940 | N/A |
| 36h9 | 13,436 | MAVA5_05210 | mycobacterial persistence regulator mrpa | Regulatory functions |
| 15b11 | 15,837 | MAVA5_05260 | acetyltransferase, GNAT family | Unknown function |
| **Mutant** | **d7/O.D.** | **A5 gene** | **Description** | **JCVI functional category** |
| 36d7 | 7,469 | MAVA5_05310 | rieske [2Fe-2S] domain protein | Energy metabolism |
| 37c1 | 12,110 | MAVA5_05580 | diaminopimelate decarboxylase | Amino acid biosynthesis |
| 16e4 | 11,954 | MAVA5_05585 | transcriptional-repair coupling factor | DNA metabolism |
| 41c1 | 7,184 | MAVA5_05585 | transcription-repair coupling factor | DNA metabolism |
| 45e4 | 6,972 | MAVA5_05645 | K+-transporting ATPase, A subunit | Transport and binding proteins |
| 31g7 | 11,779 | MAVA5_05750 | cysteine dioxygenase type I superfamily | Energy metabolism |
| 12h10 | 13,861 | MAVA5_05775 | permease of the major facilitator superfamily | Transport and binding proteins |
| 37h10 | 12,264 | MAVA5_05920 | 3-beta hydroxysteroid dehydrogenase/isomerase family | Energy metabolism |
| 39f10 | 6,872 | MAVA5_06175 | conserved hypothetical protein | Hypothetical proteins |
| 25a2 | 8,759 | MAVA5_06190 | Mcr protein | DNA metabolism |
| 33f11 | 9,850 | intergenic | 10bp upstream from MAVA5_06230 | N/A |
| 33g10 | 13,703 | MAVA5_06285 | nitrate reductase, alpha subunit | Energy metabolism |
| 18e8 | 8,853 | intergenic | 90bp upstream from MAVA5_06615 | N/A |
| 29b2 | 19,306 | intergenic | 20bp upstream from MAVA5_06690 | N/A |
| 33g2 | 10,496 | intergenic | 2bp upstream from MAVA5_06800 | N/A |
| 10e8 | 22,099 | MAVA5_06930 | StaS protein | Protein fate |
| 36h7 | 9,107 | MAVA5_06955 | TrkA-N domain family | Transport and binding proteins |
| 40h7 | 9,553 | MAVA5_07075 | gp13 protein | Mobile element functions |
| 14g4 | 8,077 | MAVA5_07205 | conserved hypothetical protein | Hypothetical proteins |
| 28g12 | 11,671 | intergenic | 23 bp upstream from MAVA5_07680 | N/A |
| 45e7 | 8,405 | MAVA5_07915 | Carboxyl transferase domain | Fatty acid and phospholipid metabolism |
| 33c10 | 10,554 | MAVA5_08200 | transcriptional regulator, TetR family protein | Regulatory functions |
| 15c6 | 13,021 | MAVA5_08465 | sulfate/thiosulfate import ATP-binding protein CysA | Transport and binding proteins |
| 36d5 | 7,616 | MAVA5_08485 | conserved hypothetical protein | Hypothetical proteins |
| 36h6 | 6,382 | intergenic | 257 bp upstream from MAVA5_08600 | N/A |
| 24b6 | 8,505 | MAVA5_08785 | conserved hypothetical protein | Hypothetical proteins |
| 27b6 | 13,623 | intergenic | 100bp upstream from MAVA5_09125 | N/A |
| 38c1 | 9,816 | MAVA5_09320 | LppO protein | Unclassified |
| 1c5 | 14,295 | MAVA5_09350 | conserved hypothetical protein | Hypothetical proteins |
| 27f8 | 11,371 | MAVA5_09405 | cytochrome P450 superfamily | Cellular processes |
| 44g4 | 10,422 | intergenic | 106bp upstream from MAVA5_09465 | N/A |
| 37c7 | 9,100 | MAVA5_09495 | conserved hypothetical protein | Hypothetical proteins |
| 18b11 | 11,722 | MAVA5_09590 | pyruvate dehydrogenase E1 component | Energy metabolism |
| 41e1 | 10,274 | MAVA5_09740 | glutamate-ammonia-ligase adenylyltransferase | Amino acid biosynthesis |
| **Mutant** | **d7/O.D.** | **A5 gene** | **Description** | **JCVI functional category** |
| 23d4 | 18,640 | MAVA5_10205 | 5,10-methylenetetrahydromethanopterin reductase | Energy metabolism |
| 40h2 | 7,581 | MAVA5_10220 | conserved hypothetical protein | Hypothetical proteins |
| 26e12 | 10,357 | MAVA5_10275 | metal-dependent hydrolase | Cellular processes |
| 29b11 | 8,659 | MAVA5_10275 | metal-dependent hydrolase | Cellular processes |
| 7d3 | 9,918 | MAVA5_10275 | metal-dependent hydrolase | Cellular processes |
| 37h6 | 9,801 | MAVA5_10295 | flavin-binding monooxygenase | Unknown function |
| 43f8 | 9,153 | MAVA5_10310 | monooxygenase | Energy metabolism |
| 5d3 | 9,876 | MAVA5_10310 | monooxygenase | Energy metabolism |
| 37d4 | 10,315 | MAVA5_10315 | cytochrome P450 | Central intermediary metabolism |
| 29e7 | 6,619 | MAVA5_10575 | ABC-transporter integral membrane protein | Transport and binding proteins |
| 1e5 | 7,871 | MAVA5_10615 | PimT protein | Unclassified |
| 41b1 | 7,313 | intergenic | 180bp upstream from MAVA5_10720 | N/A |
| 26b3 | 9,015 | MAVA5_10770 | precorrini-3B synthase | Biosynthesis of cofactors |
| 38c11 | 7,352 | intergenic | 16bp upstream from MAVA5_11070 | N/A |
| 22g7 | 6,850 | intergenic | non-CDS region. Nearest is MAVA5_11210 | N/A |
| 24c7 | 9,529 | MAVA5_11225 | oxidoreductase | Central intermediary metabolism |
| 5f6 | 9,914 | MAVA5_11355 | PapA2 protein | Cellular processes |
| 46a1 | 8,707 | MAVA5_11495 | catalase/peroxidase HPI | Cellular processes |
| 33f5 | 10,169 | MAVA5_11535 | glycosyl hydrolases family 16 | Energy metabolism |
| 29a4 | 11,193 | MAVA5_11595 | hyp. | Hypothetical proteins |
| 16g11 | 10,059 | MAVA5_11885 | antigen 85-C | Central intermediary metabolism |
| 6g5 | 17,241 | MAVA5_12060 | short chain dehydrogenase | Energy metabolism |
| 30e6 | 8,683 | intergenic | 11 bp upstream from MAVA5_12130 | N/A |
| 44b10 | 8,667 | MAVA5_12135 | transcriptional repressor, CopY family | Regulatory functions |
| 34g9 | 10,443 | intergenic | 67 bp upstream from MAVA5_12190 | N/A |
| 37e2 | 9,103 | MAVA5_12205 | glycine dehydrogenase | Energy metabolism |
| 39f5 | 8,697 | MAVA5_12405 | ppe family protein | Unknown function |
| 11c2 | 8,293 | MAVA5_12450 | 4-alpha-glucanotransferase | Energy metabolism |
| 28c4 | 8,430 | MAVA5_12985 | gnat-family acetyltransferase | Unknown function |
| 21d5 | 8,566 | MAVA5_13175 | conserved hypothetical protein | Hypothetical proteins |
| 36f9 | 7,932 | MAVA5_13185 | glucose-methanol-choline oxidoreductase | Central intermediary metabolism |
| 39d10 | 8,000 | MAVA5_13215 | P450 heme-thiolate protein | Energy metabolism |
| 7b10 | 13,985 | intergenic | 18 bp upstream from MAVA5_13345 | N/A |
| 7b5 | 14,055 | MAVA5_13410 | excinuclease ABC, B subunit | DNA metabolism |
| **Mutant** | **d7/O.D.** | **A5 gene** | **Description** | **JCVI functional category** |
| 9e11 | 19,286 | MAVA5_13430 | PknF protein | Unclassified |
| 25g7 | 8,135 | intergenic | 57 bp downstream from MAVA5_13635 | N/A |
| 42c3 | 6,654 | MAVA5_13685 | Hypothetical protein | Hypothetical proteins |
| 32d2 | 7,237 | MAVA5_13725 | adenosylmethionine-8-amino-7-oxononanoate transaminase | Biosynthesis of cofactors |
| 24b7 | 7,719 | MAVA5_13740 | putative acyltransferase domain protein | Unknown function |
| 24d3 | 7,105 | MAVA5_13900 | Hypothetical protein | Hypothetical proteins |
| 12b8 | 8,694 | MAVA5_13915 | syringomycin synthetase | Cellular processes |
| 6f4 | 11,695 | MAVA5_14420 | acyltransferase domain protein | Cell envelope |
| 35h2 | 9,269 | MAVA5_14570 | conserved hypothetical protein | Hypothetical proteins |
| 31a3 | 11,338 | intergenic | 67 bp downstream from MAVA5_14995 | N/A |
| 27c4 | 10,722 | MAVA5_15290 | conserved hypothetical protein | Hypothetical proteins |
| 39f9 | 8,372 | MAVA5_15295 | integrase | Mobile element functions |
| 14a1 | 14,085 | MAVA5_15645 | TrkA protein | Transport and binding proteins |
| 11d6 | 9,083 | MAVA5_15780 | conserved hypothetical protein | Unclassified |
| 40f8 | 7,184 | intergenic | 100 bp upstream from MAVA5_15795 | N/A |
| 7e2 | 8,024 | MAVA5_16155 | conserved hypothetical protein | Hypothetical proteins |
| 13f3 | 16,446 | intergenic | 106 bp upstream from MAVA5_16800 | N/A |
| 6c11 | 9679 | MAVA5_16900 | conserved hypothetical protein | Hypothetical proteins |
| 9d7 | 20,389 | MAVA5_17275 | carbon starvation protein A | Cellular processes |
| 30e2 | 9,158 | MAVA5_17720 | hypothetical protein | Hypothetical proteins |
| 45f9 | 8,512 | MAVA5_17915 | protein-glutamate methylesterase | Cellular processes |
| 3d8 | 9,793 | MAVA5_18030 | UDP-glucose 6-dehydrogenase | Cell envelope |
| 23e7 | 9,292 | MAVA5_18190 | hyp | Hypothetical proteins |
| 36c1 | 11,491 | intergenic | 67 bp upstream from MAVA5_18275 | N/A |
| 32h1 | 10,187 | MAVA5_18280 | ATP-dependent DNA helicase | DNA metabolism |
| 19c2 | 12,186 | intergenic | non-CDS region. Nearest is MAVA5_18430 | N/A |
| 46d2 | 11,179 | intergenic | 41 bp upstream from MAVA5_18465 | N/A |
| 30f1 | 10,740 | MAVA5_18475 | hyp | Hypothetical proteins |
| 13f6 | 8,596 | MAVA5_19045 | succinate dehydrogenase hydrophobic membrane anchor protein SdhD | Energy metabolism |
| 2e3 | 6,432 | MAVA5_19115 | conserved hypothetical protein | Hypothetical proteins |
| 44e11 | 8,961 | MAVA5_19400 | glutamate decarboxylase | Energy metabolism |
| 32e6 | 16,012 | MAVA5_19430 | conserved hypothetical protein | Unclassified |
| 5d9 | 7,737 | intergenic | 47 bp upstream from MAVA5_19495 | N/A |
| 1c3 | 14,839 | MAVA5_19910 | metal ion transporter, nramp family | Transport and binding proteins |
| 40c10 | 6,941 | MAVA5_19945 | Carbonic anhydrase | Amino acid biosynthesis |
| **Mutant** | **d7/O.D.** | **A5 gene** | **Description** | **JCVI functional category** |
| 4b8 | 10,861 | MAVA5_20270 | conserved hypothetical protein | Hypothetical proteins |
| 39e5 | 6,798 | MAVA5_20295 | cyclopropane-fatty-acyl-phospholipid synthase 2 | Fatty acid and phospholipid metabolism |
| 11d3 | 8,857 | MAVA5_20565 | glyoxalase family protein | Unknown function |
| 11c8 | 9,382 | MAVA5_20630 | cell division protein 48, N- domain | Cellular processes |
| 40e7 | 8,775 | MAVA5_20915 | O-succinylhomoserine sulfhydrylase | Amino acid biosynthesis |
| 8c10 | 10,371 | MAVA5_21435 | ATPase, AAA family | Unknown function |
| 1a2 | 8,252 | MAVA5_21480 | conserved hypothetical protein | Hypothetical proteins |
| 24g3 | 17,619 | MAVA5_21530 | acyl-CoA synthase | Fatty acid and phospholipid metabolism |
| 12g6 | 11,542 | intergenic | 119 bp upstream from MAVA5_21540 | N/A |
| 29c4 | 9,369 | MAVA5_21610 | succinate dehydrogenase | Energy metabolism |
| 29f12 | 9,305 | MAVA5_21635 | putative acyl-CoA dehydrogenase | Fatty acid and phospholipid metabolism |
| 15f7 | 11,277 | intergenic | 7 bp upstream from MAVA5_21660 | N/A |
| 4c2 | 10,781 | MAVA5_21735 | transcriptional regulator, TetR family protein | Regulatory functions |
| 26a3 | 10,085 | MAVA5_21745 | phosphotriesterase homology protein | Central intermediary metabolism |
| 20h4 | 20,692 | MAVA5_21905 | MmpL11 protein | Cell envelope |
| 38g8 | 7,037 | intergenic | 58 bp upstream from MAVA5_21955 | N/A |
| 8e7 | 9,087 | MAVA5_21960 | membrane protein | Cell envelope |
| 39c3 | 6,953 | intergenic | 58 bp upstream from MAVA5_21970 | N/A |
| 31g1 | 12,622 | intergenic | 85 bp upstream from MAVA5_22270 | N/A |
| 32a4 | 10,238 | MAVA5_22290 | transcriptional regulator, TetR family protein | Regulatory functions |
| 43f4 | 13,302 | MAVA5_22665 | TetR-family transcriptional regulator, putative | Regulatory functions |
| 20e5 | 10,005 | MAVA5_22765 | carbonic anhydrase | Cellular processes |
| 2h7 | 10,246 | MAVA5_22885 | conserved hypothetical protein, putative | Hypothetical proteins |
| 45c6 | 14,360 | MAVA5_22890 | fructose-bisphosphate aldolase class-I | Energy metabolism |
