# Supplementary material for: Identification of Bicarbonate as a Trigger and Genes Involved with Extracellular DNA Export in Mycobacterial Biofilms
Source: mBio. 2016 Dec 6;7(6):e01597-16. doi: 10.1128/mBio.01597-16 (PMC5142616; doi:10.1128/mBio.01597-16)
Supplement: Table S3 — Unique M. avium subsp. hominissuis strain A5 genomic region that nine eDNA-deficient mutants were located within. [file mbo006163096st3.docx]

Supplemental Table 3. Unique *M. avium* subsp. *hominissuis* strain A5 genomic region that nine eDNA deficient mutants were located within.

| Gene | Predicted protein |
| --- | --- |
| MAVA5_10140 | hypothetical protein |
| MAVA5_10145 | zinc-containing alcohol dehydrogenase |
| MAVA5_10150 | acetyl-CoA acetyltransferase |
| MAVA5_10155 | hypothetical protein |
| MAVA5_10160 | putative transcriptional regulator, TetR |
| MAVA5_10165 | phenylacetic acid degradation-related protein |
| MAVA5_10170 | putative hydrolase (alpha/beta fold) |
| MAVA5_10175 | TetR family transcriptional regulator |
| MAVA5_10180 | ferredoxin reductase |
| MAVA5_10185 | cytochrome P450 |
| MAVA5_10190 | ferredoxin |
| MAVA5_10195 | HTH-type transcriptional regulator AraC |
| MAVA5_10200 | hypothetical protein |
| MAVA5_10205 | luciferase-like |
| MAVA5_10210 | hypothetical protein |
| MAVA5_10215 | MaoC-like dehydratase |
| MAVA5_10220 | hypothetical protein |
| MAVA5_10225 | hypothetical protein |
| MAVA5_10230 | enoyl-CoA dehydratase/isomerase |
| MAVA5_10235 | hypothetical protein |
| MAVA5_10240 | hypothetical protein, disrupted |
| MAVA5_10245 | TetR family transcriptional regulator |
| MAVA5_10250 | acid-CoA ligase |
| MAVA5_10255 | acyl-CoA dehydrogenase FadE |
| MAVA5_10260 | acetyl/propionyl carboxylase alpha subunit |
| MAVA5_10265 | acetyl/propionyl carboxylase beta subunit |
| MAVA5_10270 | putative transcriptional regulator |
| MAVA5_10275 | hypothetical protein |
| MAVA5_10280 | putative transcriptional regulator |
| MAVA5_10285 | short-chain dehydrogenase |
| MAVA5_10290 | hypothetical protein |
| MAVA5_10295 | putative monooxygenase |
| MAVA5_10300 | putative monooxygenase |
| MAVA5_10305 | acetyl hydrolase |
| MAVA5_10310 | monooxygenase |
| MAVA5_10315 | cytochrome P450 |
| MAVA5_10320 | hypothetical protein |
| MAVA5_10325 | long-chain-fatty-acid--CoA ligase |
| MAVA5_10330 | acyl CoA dehydrogenase |
| MAVA5_10335 | hypothetical protein |
| MAVA5_10340 | hypothetical protein |
| MAVA5_10345 | zinc-type alcohol dehydrogenase AdhD |
| MAVA5_10350 | acyl CoA dehydrogenase |
| MAVA5_10355 | metal-dependent phosphohydrolase |
| MAVA5_10360 | putative MutT/NUDIX-like protein |
| MAVA5_10365 | hypothetical protein |
| MAVA5_10370 | putative regulatory protein |
| MAVA5_10375 | cell division FtsK/SpoIIIE protein |
| MAVA5_10380 | putative plasmid replication initiator protein |
