# Supplementary material for: Identification of Bicarbonate as a Trigger and Genes Involved with Extracellular DNA Export in Mycobacterial Biofilms
Source: mBio. 2016 Dec 6;7(6):e01597-16. doi: 10.1128/mBio.01597-16 (PMC5142616; doi:10.1128/mBio.01597-16)
Supplement: Table S4 — Primers used in this study. [file mbo006163096st4.docx]

Supplemental Table 4. Primers used in this Study.

| Description | Sequence (5’ to 3’) |
| --- | --- |
| Saldg (for both adapters) | TAGCTTATTCCTCAAGGCACGAGC |
| Salpt (for SalI adapter) | TCGAGCTCGTGC |
| Bampt (for BamHI/BglII adapter) | GATCGCTCGTGCC |
| pSalg R (for LMPCR reaction) | GCTTATTCCTCAAGGCACGA |
| pMyco F1 (for LMPCR reaction) | CCGGGGACTTATCAGCCAAC |
| pMyco F2 (for LMPCR reaction) | ACCCGTGATATTGCTGAAGAG |
| 11e7 MAVA5_03380 F | AAAAAAgaattcCCCGTAGTACCTAGCGATCC |
| 11e7 MAVA5_03380 R | TTTTTTaagcttGGTGTTGATGCCGAGAGTTG |
| 27c4 MAVA5_15295 F | AAAAAAgaattcCAAACGGCCTTGATGACGG |
| 27c4 MAVA5_15295 R | TTTTTTaagcttCGCTCGGCCAATATAACTCC |
| 8e7 MAVA5_21960 F | AAAAAAgaattcGACCCCAATCCCGTGCATAT |
| 8e7 MAVA5_21960 R | TTTTTTaagcttGCGACCGAAAGTGGATCAG |
| 26b3 MAVA5_10770 F | AAAAAAgaattcAGGGTTTAGGTCTGGCTGAA |
| 26b3 MAVA5_10770 R | TTTTTTgtcgacATAGATCTCCGCCGCATCG |
| 38h12 MAVA5_03425 F | AAAAAAtctagaTCGTCGTCCAAGTAAGAGCA |
| 38h12 MAVA5_03425 R | TTTTTTaagcttCGTGGTCGGCGAATTAGC |
| 26e9 MAVA5_03165 F | AAAAAAtctagaCGCCAATATTTCCGCCTACC |
| 26e9 MAVA5_03165 R | TTTTTTaagcttGCCACCGAAGAATCTGTTGA |
| 40c10 MAVA5_19945 F | AAAAAAtctagaCTTTGCGTTCTCTATGGGCC |
| 40c10 MAVA5_19945 R | TTTTTTaagcttATTGCACATCAGGATCGACG |
| 16g11 MAVA5_11885 F | AAAAAAgaattcTGAGAGCCGGAACAGACG |
| 16g11 MAVA5_11885 R | TTTTTTgtcgacGAACGGTCGAGGGGTTATCC |
| 29b11 MAVA5_10275 F | AAAAAAgaattcGGACAGCCCGCCATAAACA |
| 29b11 MAVA5_10275 R | TTTTTTgtcgacCCGCACTTCCGCATCTTTG |
